# Neuroligin1: a cell adhesion molecule that recruits PSD-95 and NMDA receptors by distinct mechanisms during synaptogenesis

**DOI:** 10.1186/1749-8104-4-17

**Published:** 2009-05-18

**Authors:** Stephanie L Barrow, John RL Constable, Eliana Clark, Faten El-Sabeawy, A Kimberley McAllister, Philip Washbourne

**Affiliations:** 1 Center for Neuroscience, University of California Davis, 1544 Newton Ct, Davis, California 95616, USA; 2 Institute of Neuroscience, University of Oregon, Eugene, Oregon 97403, USA

## Abstract

**Background:**

The cell adhesion molecule pair neuroligin1 (Nlg1) and β-neurexin (β-NRX) is a powerful inducer of postsynaptic differentiation of glutamatergic synapses *in vitro*. Because Nlg1 induces accumulation of two essential components of the postsynaptic density (PSD) – PSD-95 and NMDA receptors (NMDARs) – and can physically bind PSD-95 and NMDARs at mature synapses, it has been proposed that Nlg1 recruits NMDARs to synapses through its interaction with PSD-95. However, PSD-95 and NMDARs are recruited to nascent synapses independently and it is not known if Nlg1 accumulates at synapses before these PSD proteins. Here, we investigate how a single type of cell adhesion molecule can recruit multiple types of synaptic proteins to new synapses with distinct mechanisms and time courses.

**Results:**

Nlg1 was present in young cortical neurons in two distinct pools before synaptogenesis, diffuse and clustered. Time-lapse imaging revealed that the diffuse Nlg1 aggregated at, and the clustered Nlg1 moved to, sites of axodendritic contact with a rapid time course. Using a patching assay that artificially induced clusters of Nlg, the time course and mechanisms of recruitment of PSD-95 and NMDARs to those Nlg clusters were characterized. Patching Nlg induced clustering of PSD-95 via a slow palmitoylation-dependent step. In contrast, NMDARs directly associated with clusters of Nlg1 during trafficking. Nlg1 and NMDARs were highly colocalized in dendrites before synaptogenesis and they became enriched with a similar time course at synapses with age. Patching of Nlg1 dramatically decreased the mobility of NMDAR transport packets. Finally, Nlg1 was biochemically associated with NMDAR transport packets, presumably through binding of NMDARs to MAGUK proteins that, in turn, bind Nlg1. This interaction was essential for colocalization and co-transport of Nlg1 with NMDARs.

**Conclusion:**

Our results suggest that axodendritic contact leads to rapid accumulation of Nlg1, recruitment of NMDARs co-transported with Nlg1 soon thereafter, followed by a slower, independent recruitment of PSD-95 to those nascent synapses.

## Background

Formation of glutamatergic synapses in the central nervous system (CNS) occurs rapidly after axodendritic contact by the concurrent recruitment of mobile transport packets that contain pre- and postsynaptic proteins [1,2]. Current models suggest that formation of the presynaptic terminal occurs through recruitment of multi-protein-containing transport vesicles, while the postsynaptic density (PSD) is formed through independent recruitment of postsynaptic proteins [1-4]. Although trans-synaptic adhesion molecules induce the formation of glutamatergic CNS synapses [5,6], it is unclear when these molecules first accumulate at synapses relative to other synaptic proteins and how a single type of cell adhesion molecule (CAM) can recruit multiple types of synaptic proteins to new synapses with distinct mechanisms and time courses.

During formation of the PSD, NMDA ((N-methyl-D-aspartic acid) receptors (NMDARs) and the scaffolding protein PSD-95 are recruited to nascent synapses independently and with distinct time courses [1,7]. NMDARs are present in mobile clusters, called NMDAR transport packets (NRTPs), that are rapidly recruited to new sites of axodendritic contact, but do not colocalize with PSD-95 in dendrites before synaptogenesis [7-9]. PSD-95, a prominent scaffolding molecule in mature PSDs that binds to NMDARs [10,11], is also present in young neurons [7,8,12,13]. Although a small proportion of PSD-95 clusters are mobile, most are stationary [12-17] and accumulate at nascent synapses through coalescence from a diffuse pool [12] with a highly variable time course relative to NMDAR recruitment [7,8,17].

Formation of the glutamatergic PSD is regulated by interactions between the trans-synaptic CAMs β-neurexin (β-NRX) and neuroligin (Nlg) [6,18-22]. Although a small proportion of glutamatergic synapses can form at stable, pre-existing Nlg1 clusters associated with scaffolding molecule complexes [13], the dynamics of Nlg1 before synaptogenesis and the timing of Nlg1 accumulation at most axodendritic contacts relative to other postsynaptic proteins remains unknown. Beads or non-neuronal cells expressing β-NRX induce clustering of NMDARs and PSD-95 after 2–4 hours of contact in CNS neurons through binding to Nlg on the postsynaptic dendrite [19,20,23]. In addition, overexpression of Nlgs increases [19,20,24-28], and RNA interference knockdown of Nlgs decreases, the density of glutamatergic synapses [24]. Finally, although synapse density is not affected by knocking out Nlg1, Nlg2 and Nlg3 (either singly or in combination) in mice, neurons from these animals exhibit decreased NMDAR subunit 1 (NR1) expression and NMDAR-mediated synaptic transmission, suggesting that Nlg1 is important for recruitment of the proper amount of postsynaptic proteins to synapses [28,29].

Despite strong evidence that Nlg clustering induces differentiation of the PSD, it is unclear when Nlg1 accumulates at nascent synapses relative to first contact between axonal growth cones and dendrites and how Nlgs might subsequently recruit NMDARs and PSD-95 to new synapses independently and with distinct time courses. To address these issues, we used a Nlg-patching assay, immunocytochemistry, time-lapse imaging, and biochemical immuno-isolation of NRTPs. Nlg1 is present in two distinct pools on the surface of neurons – diffuse and clustered. Axodendritic contact leads to the rapid accumulation of Nlg1 from both pools within seconds to minutes of contact. Patching of diffuse Nlg1 aggregates PSD-95 from a cytoplasmic pool via a slow palmitoylation-dependent step. NMDARs, on the other hand, are colocalized with clustered Nlg before synapses are formed and mobile NRTPs have a high propensity to travel with Nlg. NRTPs can interact stably or transiently with Nlg during transport and patching Nlg dramatically reduces their mobility. Furthermore, both biochemical immuno-isolation and time-lapse imaging of Nlg mutants show that the association of Nlg with NRTPs occurs via interactions with PDZ domain-containing proteins such as synapse-associated protein (SAP)102 and/or synaptic scaffolding molecule (S-SCAM). Our results suggest that the formation of glutamatergic synapses proceeds by the concomitant and rapid recruitment of NMDARs with Nlg1 to a nascent synaptic contact followed by a slower, independent recruitment of PSD-95.

## Results

### Dynamics of Nlg1 in cortical neurons

**Figure 1:**
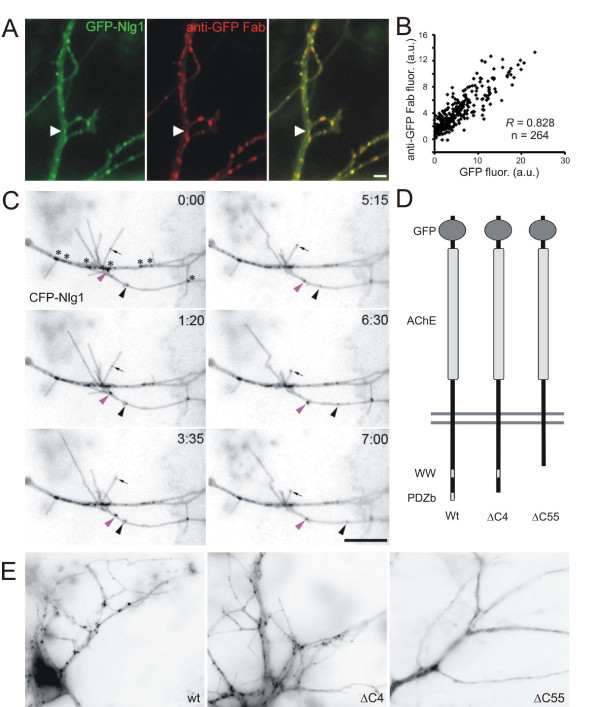
**Nlg1 clusters were mobile in dendrites of cortical neurons**. **(A) **Image of a live neuron transfected with GFP-Nlg1 (green) and surface-labeled with anti-GFP Fab fragment (red). The arrowhead denotes one of the few clusters of GFP-Nlg1 that was not labeled by the Fab fragment. Scale bar, 5 μm. **(B) **Plot of the fluorescence intensities (in arbitrary units (a.u.)) for GFP-Nlg1 and anti-GFP Fab fragment from five neurons, showing strong correlation of intensities. **(C) **Time-lapse imaging of 4 d.i.v. neurons transfected with CFP-Nlg1 revealed Nlg1 clusters that were mobile in the dendrites (pink and black arrowheads) and filopodia (arrow). Immobile clusters are indicated by asterisks in the first panel. Time is in minutes and seconds. Scale bar, 10 μm. **(D) **Diagram depicting the principle interaction domains of Nlg1 and the deletions of the carboxy-terminal 4 and 55 amino acids (ΔC4, ΔC55). AChE, acetylcholinesterase domain; PDZb, PDZ binding motif; WW, ww interaction domain; Wt, wild type. **(E) **Images of live transfected neurons demonstrated the punctate distribution of GFP-Nlg1 (wild type (wt)) and GFP-Nlg1ΔC4 (ΔC4). The localization of GFP-Nlg1ΔC55 (ΔC55) was diffuse and showed very few clusters.

Although most Nlg1 is found on the surface of young neurons [9,30], the dynamics of surface Nlg during synaptogenesis has not been described. To address this issue, cortical neurons at 4–5 days *in vitro *(d.i.v.) were transfected with green fluorescent protein (GFP)-Nlg1 (containing both a and b splice inserts) and surface-exposed GFP was labeled with anti-GFP Fab fragment (Figure 1A). Labeling with this protocol revealed a diffuse distribution of surface GFP-Nlg1, punctuated by distinct clusters. Over 99% of clusters of GFP-Nlg1 were at the surface as only 0.78% of GFP fluorescent clusters were not revealed by a 10-minute incubation with anti-GFP Fab fragment (arrowheads in Figure 1A). Furthermore, the high correlation between the GFP fluorescence and the anti-GFP Fab fluorescence (*R *= 0.828, n = 264; Figure 1B), and the lack of permeabilization of these neurons (Additional file 1E), suggest that clusters are located at the surface with little to no internal pools of Nlg1. This localization is likely to replicate that of endogenous Nlg1 since the GFP is located in the extracellular domain, making it readily accessible to antibodies while minimizing interference with intracellular effector molecules such as PSD-95 [31]. In addition, GFP-Nlg1 can bind β-NRX, can induce the formation of presynaptic terminals onto transfected non-neuronal cells [32], its expression does not adversely affect neuronal health, and its dendritic localization mimics the distribution of endogenous Nlg1 (Additional file 1A).

To examine the dynamics of Nlg1 before and during synaptogenesis, 4 d.i.v. cortical neurons were transfected with cyan fluorescent protein (CFP)- or GFP-tagged Nlg1 and time-lapse imaged 12–14 hours later. As reported in older hippocampal neurons [13], Nlg1 clusters were mobile in dendrites of young cortical neurons in culture (Figure 1C). On average, 11 ± 1.8% of GFP-Nlg1 clusters were mobile (from 6 to 21%), defined as traveling more than 2 μm during a 10 minute imaging period with movement occurring during at least 3 consecutive frames (n = 24 neurons; 5 s interframe interval). Clusters were often observed in highly active filopodia (Figure 1C), moving into and out of the end of the filopodium. At any time point during imaging, 68.1 ± 11.8% of filopodia had at least one cluster of GFP-Nlg1 within them and, interestingly, 26.1 ± 10.3% of filopodia had a cluster of GFP-Nlg1 at their tips (47 filopodia analyzed from 7 neurons). This must be specific targeting of Nlg1 to filopodial tips since a membrane-targeted GFP (farnesylated GFP) was mostly diffuse (not shown). Neurons expressing farnesylated GFP only displayed regions with increased fluorescence intensity above the mean (plus one standard deviation) of the whole filopodium in 32.6 ± 5.8% of filopodia and at the tips of 5.1 ± 3.6% of filopodia (46 filopodia analyzed from 6 neurons; *P *< 0.04).

Although the carboxy-terminal PDZ domain of Nlg1 has previously been shown not to be important for Nlg clustering or synaptic localization [33], the effects of the carboxyl terminus on Nlg1 mobility have not been reported. To characterize which protein interaction domains are important for Nlg1 mobility, carboxy-terminal GFP-Nlg1 mutants were generated that lacked either the last four amino acids (ΔC4), containing the PDZ binding motif, or the last 55 amino acids (ΔC55), containing the WW domain and PDZ binding motif (Figure 1D). Deletion of 55 amino acids from the carboxyl terminus of Nlg1 leaves 17 amino acids of the 32 amino acid motif that plays a role in targeting Nlg1 to dendrites [34]. These deletion mutants were transfected into 4–5 d.i.v. neurons and imaged 12–14 hours later. Nlg1 expression in neurons transfected with ΔC4 was punctate, consistent with previous reports [33] that the PDZ binding motif of Nlg1 is not required for localization of Nlg in clusters (Figure 1E). While the ΔC55 mutant was targeted to dendrites, it was diffusely distributed, suggesting that the carboxy-terminal 55 amino acids determine the punctate distribution of Nlg1. Given that Nlg1ΔC55 was so diffuse, cluster dynamics of this mutant could not be analyzed. In contrast, the ΔC4 mutant showed similar movement (mean velocity of 11.51 ± 2.19 μm/minute and 6.5% of clusters are mobile, n = 10 cells) to wild-type GFP-Nlg1 (11.24 ± 1.3 μm/minute, n = 11 cells, *P *> 0.5). These results suggest that an interaction through the PDZ binding motif is not necessary for Nlg1 clustering or motility.

### Accumulation of Nlg1 at sites of axodendritic contact

**Figure 2:**
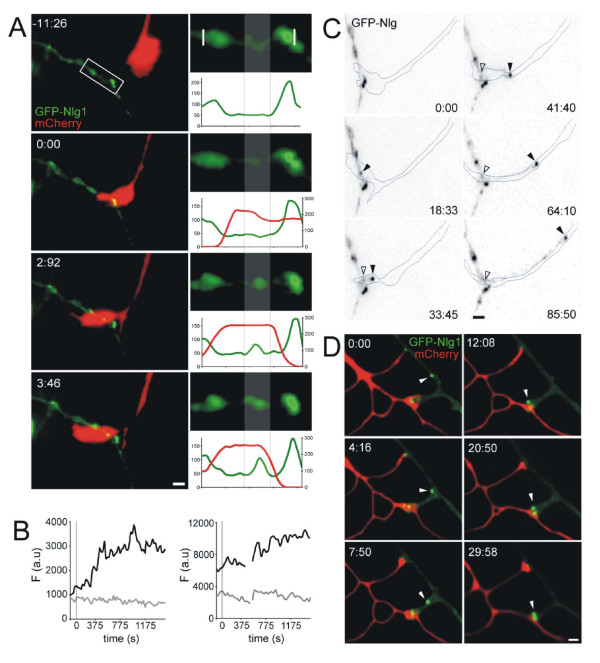
**Nlg1 accumulated at, and was transported to, sites of axodendritic contact**. **(A) **Formation of a contact between 5 d.i.v. neurons transfected with GFP-Nlg1 (green) and mCherry (red). Time 0:00 is defined as the first time of stabilized contact between the dendrite and axonal growth cone. Nlg1 accumulated at this site of contact within 3 minutes. Higher magnification images of the region of contact (white box) are shown to the right and with fluorescent intensity plots for both the dendritic (green) and axonal (red) region. **(B) **Fluorescence intensity (F) changes in arbitrary units (a.u.), for examples of rapid (left) and slower accumulation of Nlg1 (right) following contact. Black traces are taken from a dendritic segment contacted by axonal growth cones and grey traces are taken from a control dendritic segment, away from the site of contact. The gray line at time 0 represents the time of contact. Blank portions of the right trace are measurements that have been deleted due to focal drift. **(C) **Continuation of imaging of the GFP-Nlg1 transfected neuron shown in (A). Extension of a filopodium from the main dendritic branch at the site of the initial Nlg1 accumulation occurred 30 minutes following contact between the growth cone and the dendrite. Accumulation of Nlg1 in the dendrite at the site of contact (black arrowhead) clustered in the filopodial tip, which extended out over the axonal growth cone (axon shown as a dotted outline). A Nlg1 cluster remained at the tip of the filopodium during extension (open arrowhead). **(D) **Images of neighboring neurons transfected with GFP-Nlg1 (green) and mCherry (red). At time point 0:00 the axonal growth cone had already made contact with the dendrite. A cluster of Nlg1 (white arrowhead) moved to that pre-formed contact and remained stable. A stationary cluster of Nlg1, present at a second pre-existing contact site (top of image), also remained stable for the duration of imaging. Time is in minutes and seconds and the scale bars are 5 μm in all images.

Despite the general assumption that Nlgs function by accumulating rapidly at sites of axodendritic contact [20], the precise time course of Nlg1 accumulation at nascent synapses has not been reported. To address this issue, Nlg1 dynamics were examined during the formation of a stable contact between dendrites of GFP-Nlg1-transfected neurons and axonal growth cones of nearby, non-transfected neurons or neurons transfected with mCherry for visualization (Figure 2; Additional files 2 and 3). All experiments were performed between 4–6 d.i.v, a period when axodendritic contacts are frequent and synapse formation is just starting in these cultures. Images were collected every 25 s for 1–2 hours, which is the minimal interframe interval that maintains the health of the neurons while minimizing photobleaching during the long periods of imaging required to capture nascent axodendritic contacts and their stabilization.

Nlg1 accumulated at sites of axodendritic contact with a wide range of time courses that appear to fall into three categories: fast accumulation within seconds; slower accumulation over minutes; and accumulation at a site that already contained a Nlg1 cluster. Accumulation was defined by an increase in intensity of a protein cluster that was above the mean dendritic background plus one standard deviation. From the first category, GFP-Nlg1 accumulated at sites of axonal contact within an average of 75 s (n = 5 out of 9 events) after the growth cone first formed a stable contact with the dendrite. In one such example (Figure 2A), accumulation of Nlg1 occurred within 50 s (two imaging frames) after contact. Although it appears that Nlg1 accumulated at these contact sites from the diffuse pool, it is possible that we missed the rapid recruitment of a Nlg1 cluster to this site that may have occurred during the 25 s interframe interval. In a few cases, the axonal growth cone explored the dendrite and transiently touched multiple pre-existing stable GFP-Nlg1 clusters, but in all cases the axon formed a stable contact with the site at which new Nlg1 clusters appeared, as shown in Figure 2A. Slower accumulation of Nlg1 at nascent axodendritic contacts, occurring over a time-frame of minutes rather than seconds, was observed in two out of nine examples (Figure 2B). Within this second category, Nlg1 clusters accumulated at the contact within 8.1 minutes, on average, following initial contact. Although this time for arrival after contact is very similar to the time course for the recruitment of NMDAR to new synapses [7], it is possible that this average would be different if a larger number of contacts were included in our relatively modest sample. Out of all contact events observed, Nlg1 accumulated at sites of axodendritic contact within 3.03 minutes, on average (n = 7). Finally, in addition to recruitment to new synapses, Nlg1 clusters were also recruited to pre-existing axodendritic contact sites (n = 2; Figure 2D).

In several cases (four out of nine contact events), GFP-Nlg1 accumulation at sites of axodendritic contact was followed by rapid extension of a filopodium from the main dendritic branch along the contacting axon. In each of these cases, a Nlg1 cluster moved with the filopodium, clustered at its tip (Figure 2C). These filopodia eventually stopped growing along the axon and remained stationary at this site with no further filopodial extension for the remainder of imaging (for up to 2 hours). Overall, our results suggest that Nlg1 can be recruited to sites of contact with axons either rapidly, within seconds, or more slowly, within minutes. Although it has been assumed that Nlg1 accumulation is one of the first events in synaptogenesis, our results are the first direct demonstration that the time course of Nlg1 accumulation at sites of axodendritic contact is rapid enough to support the hypothesis that it is critical for inducing formation of the PSD.

### Patching of Nlg

Because the probability of observing the formation of stable axodendritic contacts between cultured neurons is prohibitively low, an alternative method was developed to aggregate surface Nlg in a rapid and reliable manner to investigate whether rapid clustering of Nlg1 induces accumulation of postsynaptic proteins. This method consists of a patching assay using a soluble ligand to cluster endogenous Nlgs or recombinant Nlg1. While this assay cannot precisely mimic the interactions of Nlg with its presynaptic ligand β-NRX on a contacting axon, it does cause Nlg aggregation through interactions with β-NRX on naïve sections of dendrite, which has been proposed to be one of the initial steps in synaptogenesis [20]. Moreover, this assay has the advantage of studying the downstream events following the aggregation of Nlgs, which may be only one of many molecular interactions that occur at axodendritic contacts. A similar method was used successfully to demonstrate the sufficiency of β-NRX, the presynaptic ligand of Nlgs, in recruiting synaptic vesicles [25]. We used cultured, cortical neurons for these experiments at 3–5 d.i.v. because they are well-differentiated but have few synapses on their dendrites at these ages [7] and thus present a naïve substrate for artificially induced Nlg1 patching.

**Figure 3:**
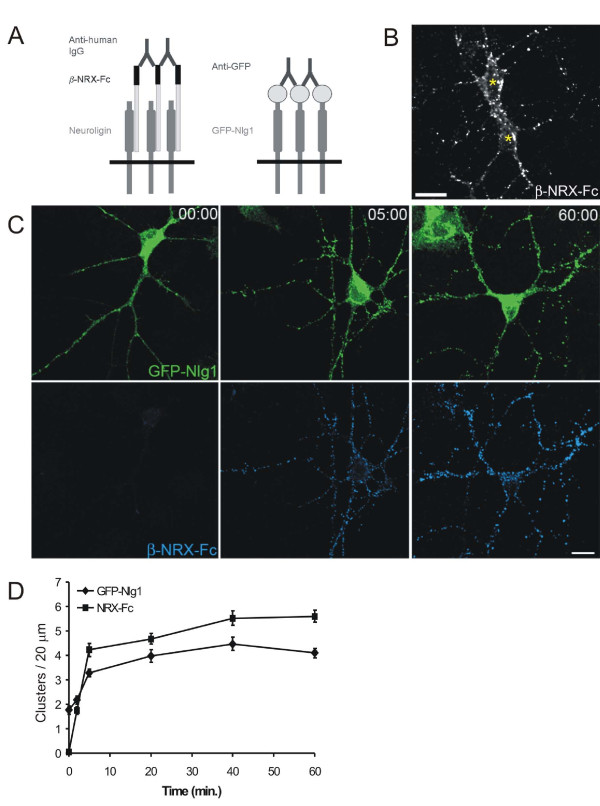
**Patching of endogenous and recombinant Nlg**. **(A) **Diagram depicting the two methods of patching: pre-oligomerized β-NRX-Fc fusion proteins were used to patch endogenous Nlg and anti-GFP antibodies to patch recombinant GFP-Nlg1. **(B) **Fluorescence image of live cortical neurons demonstrating β-NRX-Fc bound to non-transfected neurons (asterisks indicate cell bodies). Scale bar, 20 μm. **(C) **Confocal images of cortical neurons transfected with GFP-Nlg1 and immunostained with antibodies against GFP (green) and human Fc (blue) after 0, 5 and 60 minutes of patching with β-NRX-Fc. Scale bar, 20 μm. **(D) **Quantification of the time course of patching for both β-NRX-Fc clusters and GFP-Nlg1 clusters per 20 μm of dendrite (n ≥ 13 neurons for each time point).

To patch Nlg, neurons were incubated with an oligomerized β-NRX1-human IgG Fc fusion protein (β-NRX-Fc; Figure 3A). This fusion protein bound to the surface of both non-transfected and GFP-Nlg1-transfected neurons (Figure 3B, C), presumably through binding to endogenous and recombinant Nlg1 since this treatment caused diffuse GFP-Nlg1 to become patched (Figure 3C). After 60 minutes of patching, 76.7 ± 2.1% of GFP-Nlg1 clusters were colocalized with β-NRX-Fc (n = 26 neurons). Fixation and labeling of neurons at various times after addition of oligomerized β-NRX-Fc demonstrated that binding of β-NRX-Fc to neuronal membranes was rapid (Figure 3C, D). Patching of GFP-Nlg1 reached 73% of maximum within the first 5 minutes of incubation (Figure 3D) and endogenous Nlg was patched in non-transfected cells with a similar time course (data not shown).

Given that β-NRX1 can bind to Nlgs 1, 2, 3 and 4, and to multiple Nlg1 splice forms [19,35], recombinant Nlg1 was specifically clustered by transfecting neurons with GFP-Nlg1 and incubating these live neurons with an oligomerized antibody to GFP (Figure 3A). Using this approach, GFP-Nlg1 was clustered with a similar time course to treatment with β-NRX-Fc (Additional file 1B, C). Approximately 66.5% of diffuse, surface GFP-Nlg1 is brought into new patches or added to existing clusters, because the mean intensity of diffuse GFP-Nlg1 decreased from 5.8 ± 0.7 to 1.9 ± 0.2 fluorescence units between control and patched neurons (*P *< 0.0001, n = 7). The patched distribution of Nlg1, using both methods, resembled the distribution of Nlg1 in more mature neurons at a time when many synapses have been established (10 d.i.v. and later; see below).

### Patching Nlg dramatically increases PSD-95 clustering

**Figure 4:**
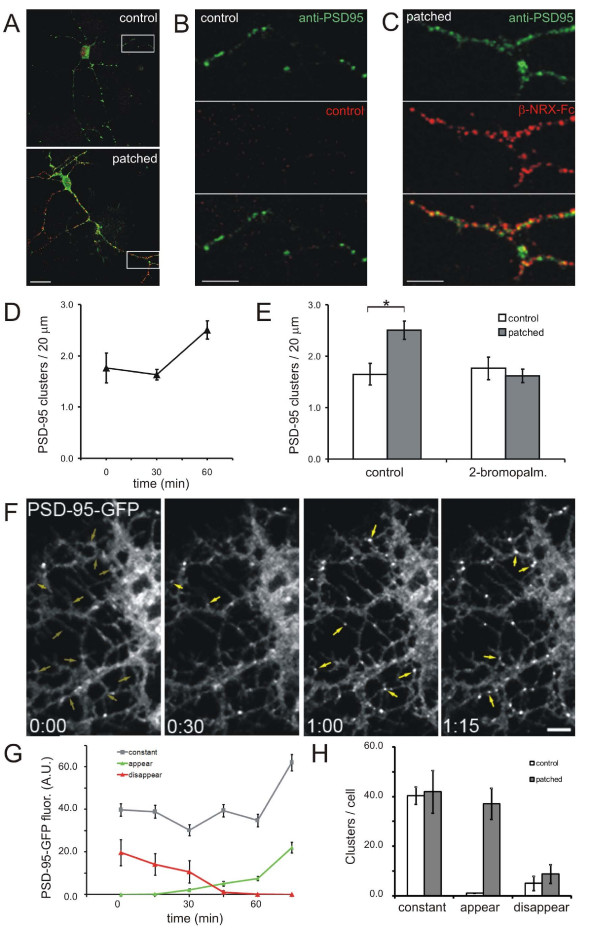
**Patching endogenous Nlg induced clustering of PSD-95**. **(A) **Patching with β-NRX-Fc (patched) increased the density of PSD-95 clusters as shown by confocal images of neurons that were incubated with β-NRX-Fc (red) for 1 hour then fixed and immunostained for PSD-95 (green). Secondary antibody only was used as the control. Scale bar, 20 μm. **(B, C) **Enlargements of the boxed regions in (A), showing control and patched dendrites, respectively. Scale bar, 10 μm. **(D) **Time-course of PSD-95 clustering by β-NRX-Fc demonstrates that the increase in PSD-95 cluster density occurred after 30 minutes of patching. **(E) **The density of PSD-95 clusters (per 20 μm of dendrite) was increased by patching Nlg, but not in the presence of 2-bromopalmitate (*P < 0.004). **(F) **Live imaging of a cortical neuron during patching with β-NRX-Fc demonstrates the appearance of new PSD-95-GFP clusters (yellow arrows). Sites at which clusters appeared are denoted with ghost arrows in the first image. Time is in hours and minutes. Scale bar, 5 μm. **(G) **Time-course of fluorescence intensities from five neurons treated with β-NRX-Fc. The locations at which intensities were measured have been categorized into sites with stable fluorescence (constant), sites at which clusters appear (appear) and sites at which clusters disappear (disappear). **(H) **Analysis of the numbers of clusters per neuron as categorized in (G). Error bars are standard error.

To determine if Nlg patching leads to accumulation of postsynaptic proteins as it has been proposed to do during the formation of the PSD, the distribution of PSD-95 before and after patching endogenous Nlgs was examined. Incubation of 3–5 d.i.v. cortical neurons with oligomerized β-NRX-Fc for 1 hour increased the density of PSD-95 clusters by about 66% (from 1.65 ± 0.15 clusters/20 μm of dendrite to 2.5 ± 0.15, n = 16 neurons, *P *< 0.004; Figure 4A–E). Many PSD-95 clusters (56.7 ± 2.6%) colocalized with β-NRX-Fc patches (Figure 4C). This is a 75% increase over the number of PSD-95 clusters that colocalized with β-NRX-Fc when β-NRX-Fc was applied after neurons were fixed (32.3 ± 4.1%, n = 16 neurons, *P *< 0.0001). The time course of PSD-95 clustering lagged considerably behind the rapid induction of Nlg patches (Figure 3D), since no effect on PSD-95 cluster density was seen at 30 minutes (1.76 ± 0.18 clusters/20 μm to 1.63 ± 0.17, n = 16 neurons, *P *> 0.5; Figure 4D). However, PSD-95 accumulation at Nlg patches reached its maximum between 30 minutes and 1 hour. This time course is generally consistent with experiments in cultured hippocampal neurons incubated with β-NRX-Fc-coated beads [23].

The delay in PSD-95 clustering after Nlg patching indicates that PSD-95 is not recruited immediately through a constant and direct interaction with Nlg. Our results suggest instead that PSD-95 accumulation at Nlg patches is mediated by an intermediary step, perhaps an enzyme-based signaling mechanism. Due to the fact that PSD-95 requires palmitoylation of its two amino-terminal cysteine residues for synaptic localization [36], we next tested if palmitoylation influences the ability of PSD-95 to be recruited to Nlg clusters. Inhibition of palmitoylation with 2-bromopalmitate (15 μM for 12 hours), completely blocked the induction of additional PSD-95 clusters by Nlg patching (1.77 ± 0.53 clusters/20 μm dendrite to 1.62 ± 0.1, n = 17 neurons, *P *> 0.18; Figure 4E). While it is important to point out that a large number of synaptic proteins are palmitoylated [37], our data are consistent with the interpretation that the accumulation of PSD-95 at Nlg patches is a palmitoylation-dependent step, similar to the accumulation of PSD-95 at synapses [36].

Next, the dynamics of Nlg-induced PSD-95 clustering was determined using time-lapse imaging. Young cortical neurons (4 d.i.v.) were transfected with PSD-95-GFP and, after 12 hours, the coverslip was mounted on a confocal microscope and neurons were incubated with oligomerized β-NRX-Fc to cluster endogenous Nlgs. Images acquired over the subsequent 75 minutes revealed the appearance of new PSD-95-GFP clusters (Figure 4F). This extended time course was chosen as these experiments were conducted at room temperature, while in previous experiments patching was performed at 37°C. Analysis of fluorescence intensities at locations where clusters were present at any time during imaging allowed us to divide the locations into four categories: locations at which clustered GFP was present during the entire imaging period (constant); locations at which clusters were not present at the beginning of the experiment (appear); locations at which clusters were not present in the last image of the series (disappear); and locations at which clusters appeared and then disappeared during the imaging period (Figure 4G). This last category represented only 4.7% of locations and was not further evaluated.

Comparison of PSD-95-GFP-transfected neurons incubated with oligomerized β-NRX-Fc and transfected neurons incubated only with secondary antibody demonstrated that the number of clusters that appeared was increased almost 40-fold by patching of Nlg (from 1 cluster per cell to 37.2 ± 6.24, n = 5 neurons per condition, *P *< 0.02; Figure 4H). In contrast, the number of constant and disappearing clusters was not affected by Nlg patching with β-NRX-Fc (*P *> 0.5; Figure 4H). Thus, live-imaging experiments confirm that binding of β-NRX is sufficient to initiate the formation of *de novo *PSD-95 clusters with a relatively slow time course of about 1 hour.

### Nlg1 is sufficient to induce PSD-95 clustering

**Figure 5:**
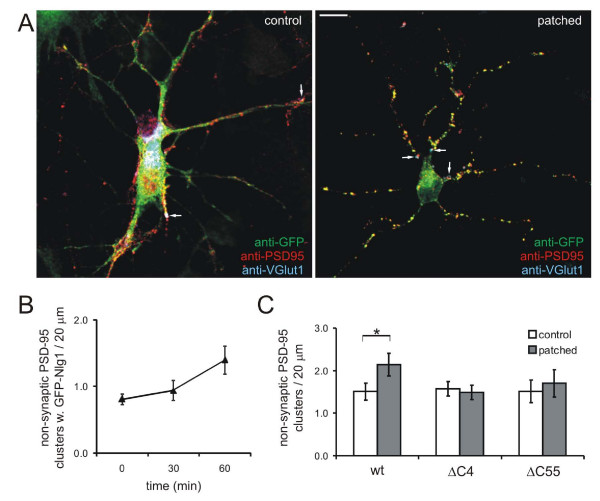
**Patching of GFP-Nlg1 induced clustering of PSD-95**. **(A) **Patching of GFP-Nlg1 with anti-GFP (green) increased the number of PSD-95 clusters in the dendrites of transfected cortical neurons (red). 'True' synapses are defined as colocalized VGlut1 (blue) and PSD-95 (red) protein clusters. Scale bar, 20 μm. **(B) **The density of non-synaptic PSD-95 clusters colocalized with Nlg1 is increased by patching recombinant GFP-Nlg1 at 60 minutes. **(C) **When synaptic clusters are excluded from analysis (by eliminating clusters that are positive for VGlut1), it is clear that the increase in PSD-95 clusters when patched with anti-GFP antibody was due to accumulation at non-synaptic sites. This clustering of PSD-95 was blocked when the carboxy-terminal region is deleted in either ΔC4 or ΔC55 mutants (*P < 0.02). Error bars are standard error.

Due to the possibility of β-NRX1 binding to multiple Nlgs in non-transfected neurons [19,35], we examined whether specifically patching recombinant GFP-Nlg1 with an anti-GFP antibody is sufficient to induce PSD-95 clustering. Treatment of GFP-Nlg1-transfected neurons with oligomerized anti-GFP antibody increased the density of PSD-95 clusters by 31% (*P *< 0.02; Figure 5A, B). To test whether Nlg1 patching caused the formation or disassembly of 'true' synapses, neurons were immunostained with antibodies to the vesicular glutamate transporter 1 (VGlut1) to label glutamatergic, presynaptic terminals, PSD-95 to label the PSD, and GFP to visualize patched GFP-Nlg1; synapses were defined as colocalized sites of VGlut1 and PSD-95 [7]. The density of presynaptic terminals or synaptically localized PSD-95 clusters was not changed after Nlg1 patching (1.3 ± 0.16 VGlut1 clusters/20 μm before and 1.39 ± 0.2 after, and 0.032 ± 0.006 synaptic PSD-95 clusters/20 μm before to 0.029 ± 0.005 after; n = 13, *P *> 0.4). However, the density of non-synaptic PSD-95 clusters increased by 42% after 1 hour of patching with anti-GFP antibodies (1.5 ± 0.11 clusters/20 μm to 2.14 ± 0.21, n = 13, *P *< 0.02; Figure 5B). Many PSD-95 clusters colocalized with the patched GFP-Nlg1 (63.5 ± 5.5%). Patching of endogenous Nlgs and recombinant Nlg1 caused similar effects on recruitment of PSD-95, indicating that GFP-Nlg1 overexpression did not cause this result. These data suggest that Nlg1 patching induces accumulation of PSD-95 primarily at extrasynaptic sites, as expected due to the nature of the patching assay. Overall, these results demonstrate that patching of Nlg1 is sufficient to cluster PSD-95 within 1 hour.

PSD-95 binds to the carboxy-terminal PDZ binding motif of Nlg1 through its third PDZ domain [31]. To examine whether this direct interaction is required for Nlg-induced PSD-95 clustering, carboxy-terminal GFP-Nlg1 mutants ΔC4 and ΔC55 were patched in 4–5 d.i.v. cortical neurons (Figure 1D). Deletion of half of the intracellular domain of Nlg1, including the PDZ binding motif and the WW interaction domain (ΔC55), blocked the effect of Nlg patching on clustering of PSD-95 (1.51 ± 0.17 clusters/20 μm, n = 13, *P *> 0.5; Figure 5C). Deletion of just the last four amino acids (ΔC4), the PDZ binding motif of Nlg1, also abrogated the effect of Nlg1 patching on PSD-95 clustering (1.57 ± 0.14 clusters/20 μm, n = 13, *P *> 0.9; Figure 5C). Thus, the ability of Nlg1 to cluster PSD-95 specifically requires the PDZ binding domain of Nlg1.

### Patching Nlg1 does not affect NMDA receptor distribution

**Figure 6:**
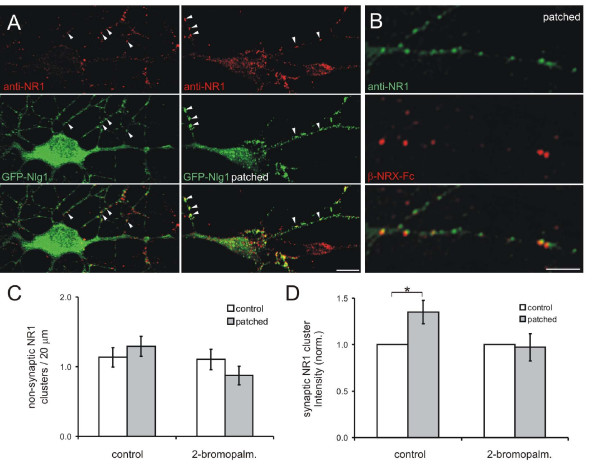
**Patching Nlg1 did not affect non-synaptic NMDA receptor distribution**. **(A) **The localization and density of NR1 clusters (red) was not altered by patching of transfected GFP-Nlg1 (green). NR1 was colocalized with GFP-Nlg1 clusters before and after patching (arrowheads). Scale bar, 10 μm. **(B) **Patching with β-NRX-Fc (red) also did not alter the already high colocalization of Nlg with NR1 (green). Scale bar, 5 μm. **(C) **Patching with β-NRX-Fc did not significantly change the density of non-synaptic NR1 clusters in cortical neurons or neurons treated with 2-bromopalmitate. **(D) **Patching of Nlg1 increased the intensity of NR1 at synaptic sites (*P < 0.05) and this increase was prevented by 2-bromopalmitate. Error bars are standard error.

Given that PSD-95 binds directly to NMDARs [10,11], Nlg1 could recruit NMDARs to new synapses via the interaction with PSD-95. To test if Nlg patching clustered NMDARs in a similar fashion as it did PSD-95, young cortical neurons (3–5 d.i.v.) were immunostained with an antibody against NR1 before and after 1 hour of patching with either β-NRX-Fc or anti-GFP antibodies. Surprisingly, patching of endogenous Nlgs or GFP-Nlg1 did not significantly change the overall density of NR1 clusters (1.66 ± 0.24 clusters/20 μm to 2.27 ± 0.36, n = 16 neurons, *P *> 0.15; Figure 6A, B) or the density of non-synaptic NR1 clusters (1.13 ± 0.14 clusters/20 μm to 1.29, n = 16, *P *> 0.4; Figure 6C; non-synaptic was defined as NR1 that did not colocalize with the synaptic vesicle protein VGlut1). Thus, in contrast to its effects on clustering of PSD-95, Nlg patching does not alter the distribution of NMDARs.

Rather than causing the formation of *de novo *accumulations of NR1, it is possible that Nlg patching might cause additional accumulation of NR1 at pre-existing NR1 clusters. Consistent with this idea, Nlg1 patching caused an increase in the fluorescence intensity of NR1 clusters, but only at synaptic locations (35.2 ± 12.5%, n = 20 neurons, *P *< 0.05; Figure 6D; synaptic NR1 is defined as NR1 clusters colocalized with vGlut1). This increase was blocked by incubation with the palmitoylation inhibitor 2-bromopalmitate (Figure 6D). Thus, Nlg1 patching recruits additional NMDARs to existing synapses via a palmitoylation-dependent step.

### Colocalization of NMDA receptors and Nlg1

**Figure 7:**
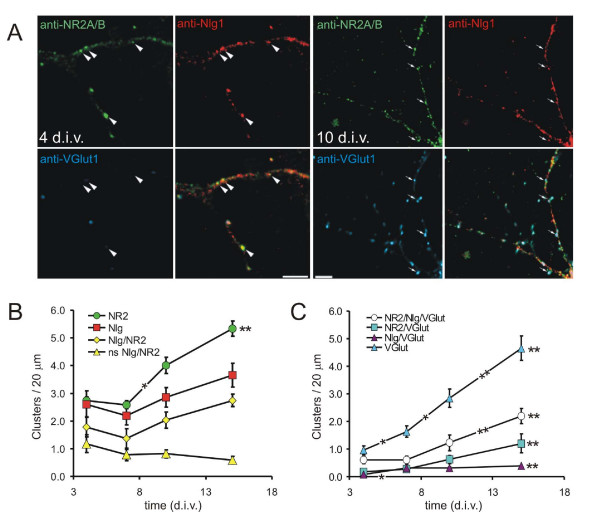
**Colocalization of Nlg1 and NMDA receptors prior to synapse formation**. **(A) **Immunostaining of cortical neurons in culture at 4 and 10 d.i.v. with antibodies to Nlg1 (red), NR2A/B(green) and VGlut1 (blue). At 4 d.i.v., the majority of colocalized clusters of Nlg1 and NR2A/B were non-synaptic (arrowheads), whereas in 10 d.i.v. neurons the majority of colocalized NR2A/B and Nlg1 were found with the presynaptic marker VGlut1 (arrows). **(B) **The density of protein clusters as indicated in the legend are plotted over time in culture. Nlg/NR2 includes the clusters of NR2 (green circles) and Nlg1 (red squares) that colocalize with each other. The rest of the cluster densities in (B, C) are plotted similarly. Clusters of colocalized NR2A/B and Nlg1 were abundant at early time points in culture. The density of non-synaptic (ns) Nlg/NR2 positive clusters decreased with time. **(C) **The densities of presynaptic terminals and presynaptic terminals with Nlg1 and NR2 increased steadily over time in culture. Error bars are standard error. *P < 0.05, **P < 0.001; asterisks at the end of the curve refer to comparisons between 4 and 15 d.i.v.

On close examination, a large proportion of both non-synaptic and synaptic NR1 was clearly colocalized with patches of GFP-Nlg1 (56.2 ± 8.4%, n = 11 neurons; Figure 6A, B). In contrast to PSD-95, colocalization of NR1 and Nlg1 was also apparent for clusters of GFP-Nlg1 before patching (51.3 ± 9.3%; arrowheads in Figure 6A, left panels), suggesting that there might be a physical interaction between Nlg1 and NMDARs prior to and during synapse formation. To test this possibility, cultured cortical neurons were immunostained with antibodies specific to Nlg1, NMDAR subunits 2A and 2B (NR2A/B) and VGlut1 at ages ranging from 4–15 d.i.v. (Figure 7A). Nlg1 was found in dendrites of all pyramidal neurons at all ages examined in distinct clusters clearly visible above a diffuse background. The total density of Nlg1 clusters did not significantly increase between 4 and 15 d.i.v. (2.61 ± 0.5 to 3.65 ± 0.4 clusters/20 μm dendrite, n = 10 neurons/time point, *P *> 0.12; Figure 7B). In contrast, the number of VGlut1-positive presynaptic terminals found on dendrites, which was very low at 4 d.i.v. (0.96 ± 0.2 clusters/20 μm dendrite), increased almost 5-fold by 15 d.i.v. (4.65 ± 0.45 clusters/20 μm dendrite, n = 10 neurons/time point, *P *< 0.00001; Figure 7C). These results indicate that clusters of Nlg1 are present in dendrites before the majority of synapses have formed, similar to a recent report showing the presence of Nlg1 clusters at both synaptic and non-synaptic sites in hippocampal neurons at older ages [13].

Consistent with our hypothesis, the majority of Nlg1 clusters colocalized with NR2A/B (Figure 7A); 66.5% ± 6.0 of Nlg1 clusters colocalized with NR2A/B at 4 d.i.v., well before the peak of synaptogenesis (between 7 and 10 d.i.v.). Surprisingly, this percentage did not significantly increase during development *in vitro *(76.6 ± 3.9% at 15 d.i.v., *P *> 0.2, n = 9 neurons/time point). NR2A/B clusters also showed no increase in the extent of colocalization with Nlg1 over time in culture, varying from 62.4 ± 8.3% at 4 d.i.v. to 50.5 ± 5.5% at 10 d.i.v. (*P *> 0.4, n = 9 neurons/time point). Yet, the number of clusters of Nlg1 and NR2A/B in close apposition to VGlut1-positive terminals increased almost 4-fold between 4 and 15 d.i.v. (0.6 ± 0.12 to 2.19 ± 0.21 clusters/20 μm dendrite, *P *< 0.00001, n = 9 neurons/time point; Figure 7C). Concomitantly, the proportion of colocalized NR2/Nlg1 clusters at non-synaptic locations decreased from 60.7% ± 7.0 at 4 d.i.v. to 20.6% ± 3.9 at 15 d.i.v (n = 9 neurons/time point, *P *< 0.0005; Figure 7B). These data suggest that endogenous Nlg1 and NMDARs are often colocalized prior to synapse formation and are either recruited to postsynaptic terminals together or with similar kinetics.

### Transport of Nlg1 with NMDA receptors

**Figure 8:**
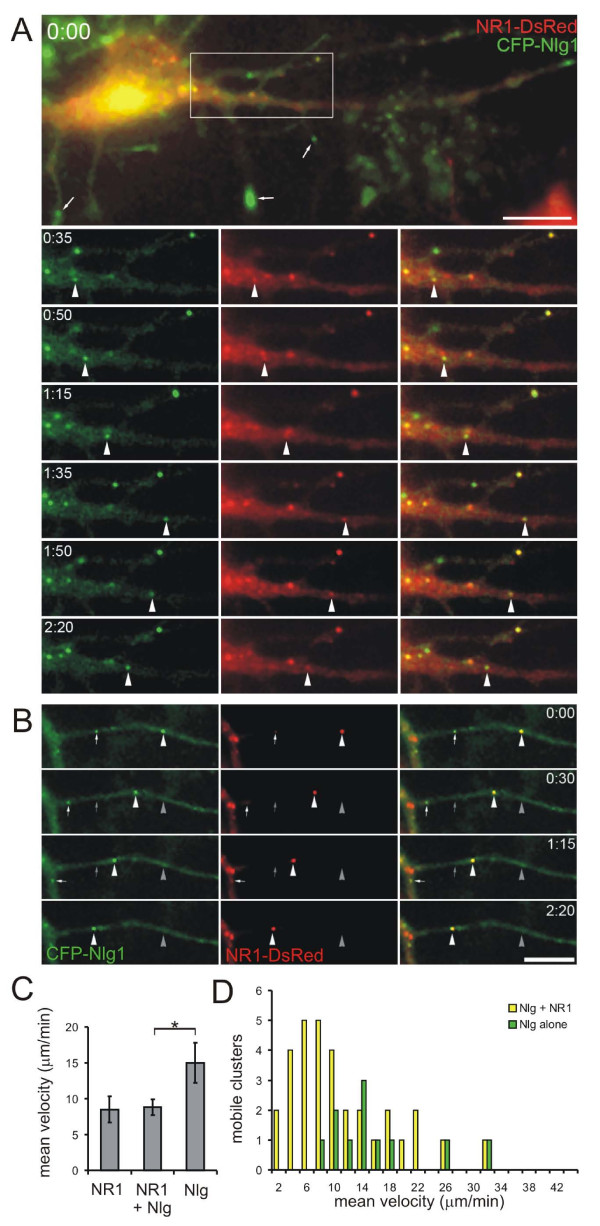
**NMDA receptor transport packets (NRTPs) and Nlg1 clusters were co-transported prior to synaptogenesis**. **(A) **Time-lapse imaging of neurons cotransfected with CFP-Nlg1 (green) and NR1-DsRed (red) demonstrates movement of CFP-Nlg1 with NR1-DsRed (arrowheads) in both the anterograde and then retrograde direction. In the first panel, CFP-Nlg1 was seen at the tips of filopodia (arrows). Lower panels are enlargements of the boxed region in the upper panel. Time is in minutes and seconds. Scale bar, 10 μm. **(B) **Time-lapse images demonstrate that CFP-Nlg1 can move with (arrowheads) and without (arrows) NR1-DsRed. Grey arrows and arrowheads indicate original locations of clusters from first image. Scale bar, 10 μm. **(C) **CFP-Nlg1 clusters had higher mean velocities than NRTPs or co-transported NR1 and Nlg1. Error bars are standard error. Only mobile clusters were included in this analysis (*P < 0.02). **(D) **Comparison of the distribution of mean velocities of mobile clusters of Nlg alone (green) and colocalized CFP-Nlg1 and NR1-DsRed (yellow).

The high colocalization of Nlg1 with NMDARs suggested that these proteins may be recruited together to nascent synapses, perhaps as mobile clusters seen in previous experiments (Figures 1C and 2) [7,9]. If so, then Nlg1 and NMDARs should be trafficked together in dendrites of cultured cortical neurons before synaptogenesis. To examine whether Nlg1 clusters are trafficked with NMDARs, 4–5 d.i.v. cortical neurons were transfected with both CFP-Nlg1 and NR1-DsRed, and imaged 12–14 hours later (Figure 8A, B; Additional file 4). As predicted from immunostaining, the majority of CFP-Nlg1 and NR1-DsRed clusters were colocalized: 53.9 ± 6.8% of CFP-Nlg1 was colocalized with NR1-DsRed, while 63.7 ± 8.0% of NR1-DsRed was colocalized with CFP-Nlg1. Time-lapse fluorescence imaging revealed that 69.7 ± 9.5% of mobile CFP-Nlg1 clusters were colocalized with NR1-DsRed, while 76.1 ± 10.4% of mobile NR1-DsRed (NRTPs) were colocalized with CFP-Nlg1 (a total of 188 clusters analyzed from 11 transfected cells). Thus, the majority of NRTPs are co-transported with clusters of Nlg1.

**Figure 9:**
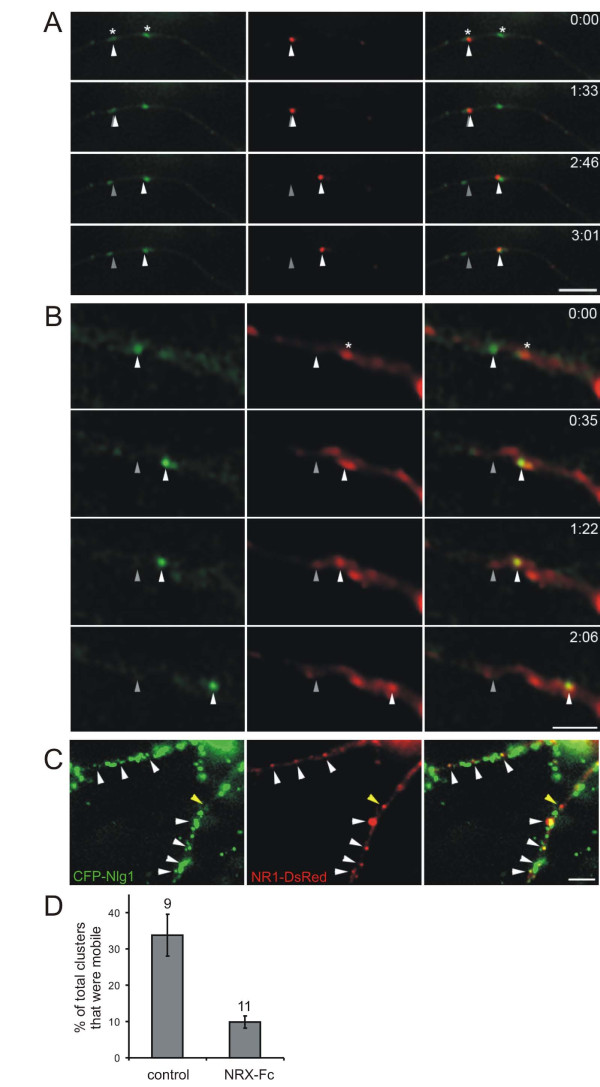
**Transient associations of Nlg and NMDA receptor transport packets (NRTPs) and the effects of Nlg patching on NRTP mobility**. **(A) **Time-lapse imaging of 4 d.i.v. neurons cotransfected with NR1-DsRed (red) and CFP-Nlg1 (green) demonstrates that NRTPs (arrowheads) can move between stable Nlg clusters. Grey arrowheads indicate original location of cluster. Clusters that were immobile during the imaging period are indicated by an asterisk in the top panel. Time is in minutes and seconds. Scale bar, 5 μm. **(B) **Alternatively, CFP-Nlg1 can move to a stable NR1-DsRed cluster and then the two can move together (arrowheads). Clusters that were immobile during the imaging period are indicated by an asterisk in the top panel. Scale bar, 5 μm. **(C) **Time-lapse imaging of neurons co-transfected with CFP-Nlg1 (green) and NR1-DsRed (red) after patching with β-NRX-Fc for at least 1 hour. Over 65% of NR1-DsRed was colocalized with CFP-Nlg1 (white arrowheads). One cluster of NR1-DsRed alone is highlighted with a yellow arrowhead. Scale bar, 10 μm. **(D) **The percentage of total NR1-DsRed clusters that were mobile was significantly decreased after patching with β-NRX-Fc (numbers of neurons analyzed are indicated above bars). Error bars are standard error.

Analysis of the kinetics of NRTPs and Nlg1 revealed two distinct pools of mobile Nlg clusters: clusters containing both NR1 and Nlg1 moved at a mean velocity of 8.8 ± 1.1 μm/minute, a speed that closely resembles our previous observations for NRTPs (8.5 ± 1.8 μm/minute; Figure 8C) [9]. In contrast, clusters of Nlg1 alone moved at a mean velocity of around 14.9 ± 2.8 μm/minute (n = 10 neurons, *P *< 0.02; Figure 8C, D). Detailed analysis of time-lapse movies revealed that these two pools of Nlg are dynamic, that is, the association between Nlg and NRTPs was occasionally transient. In 5 out of 188 clusters analyzed, we noticed NR1-DsRed clusters moving between immobile CFP-Nlg1 clusters (Figure 9A). In addition, clusters of CFP-Nlg1 moved to join a cluster of NR1-DsRed and then moved together with that NRTP (2 of 188 clusters; Figure 9B). Within the same neuron, it was possible to see NR1- plus Nlg1-positive clusters move at mean velocities of 6.5 μm/minute (n = 4), while two Nlg-only clusters averaged 12.8 μm/minute. Thus, NRTPs and Nlg clusters are not always tightly associated during movement and Nlg1 may use a different mode of transport when not associated with NMDARs. Overall, these data suggest a significant, but labile, interaction between NRTPs and Nlg1.

### Nlg patching dramatically decreases Nlg and NRTP mobility

To investigate the effect of Nlg patching on the motility of NRTPs, 4–5 d.i.v. cortical neurons were cotransfected with CFP-Nlg1 and NR1-DsRed. Twelve to 14 hours later, transfected neurons were incubated with oligomerized β-NRX-Fc for between 60 and 80 minutes prior to time-lapse fluorescence microscopy (Figure 9C). Control transfected neurons were incubated with secondary antibody only. Analysis of time-lapse movies revealed that Nlg1 patching caused a 6.25-fold reduction in the numbers of mobile CFP-Nlg1 (from 11 ± 1.8% to 1.76 ± 0.4%, n = 11 neurons, *P *< 0.002). This effect was paralleled by a 3.4-fold reduction in the percentage of mobile NR1-DsRed clusters (from 33.7 ± 5.7% to 9.85 ± 1.6%, n = 11 neurons, *P *< 0.0002; Figure 9D). This decrease in mobility of NRTPs suggests that the normally highly mobile fraction of NMDAR clusters is immobilized at clusters of surface Nlg1 by patching.

### Biochemical association of Nlg with NRTPs

**Figure 10:**
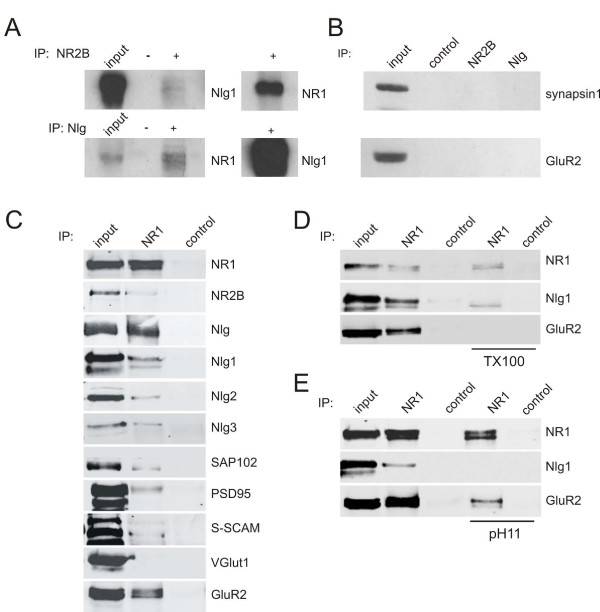
**Biochemical analysis of the interaction between Nlg and NMDA receptor-containing immuno-isolates**. **(A) **NR2B and Nlg were immunoprecipitated from detergent solubilized P4 rat visual cortex and blotted with antibodies specific for NR1 and Nlg1. NR1 and Nlg1 were co-immunoprecipitated using these antibodies, but not by Protein-G sepharose beads alone (control). **(B) **Immunoprecipitations (IPs) from detergent-solubilized P4 rat visual cortex using antibodies to NR2B and Nlg did not co-immunoprecipitate the synaptic proteins synapsin1 and GluR2. **(C) **NR1-containing membrane fractions were immuno-isolated from P2–3 rat cortex. Western blots of these organelles reveal enrichment for many other postsynaptic proteins, including Nlgs 1, 2 and 3. Input and control lanes were included for comparison. Control lanes represent isolations performed without primary antibody to NR1 (beads were coated with control IgG). Comparison of NR1 and control lanes shows enrichment of the blotted protein in NMDA receptor transport packets (NRTPs). **(D) **Nlg1 remained associated with NR1 in the immuno-isolated vesicle fraction after solubilization with Triton X-100, while GluR2 is no longer recovered. **(E) **Nlg1 was not enriched after washing with a carbonate buffer at pH11, which removes associated, but not integral vesicle proteins. In comparison, GluR2 remains associated with NRTPs under high pH conditions.

Given the colocalization and co-transport of Nlg clusters with NRTPs, we next investigated the biochemical nature of their interaction. First, we determined if Nlg and NMDARs bound one another in early postnatal cortex before the bulk of synaptogenesis occurs. Consistent with this idea, antibodies to both NR2B and Nlgs co-immunoprecipitated Nlg1 and NR1, respectively, from solubilized membranes from P4 rat cortices (Figure 10A). The interaction between Nlg1 and the NMDAR was specific because the synaptic vesicle protein synapsin1 and the AMPA (α-amino-3-hydroxy-5-methyl-4-isoxazolepropionic acid) receptor (AMPAR) subunit GluR2 were not co-immunoprecipitated with these antibodies (Figure 10B) and extended exposures of these western blots did not result in positive labeling of immunoprecpitations with either the Nlg or NR2B antibodies (not shown).

This biochemical association could potentially occur between Nlg1 and NMDARs at non-synaptic sites or at the few synapses already present in early postnatal cortex. To determine if Nlg1 binds specifically to NRTPs, NMDAR-containing isolates were prepared from a light membrane fraction from P2–3 rat cortex [38,39] using an anti-NR1 antibody conjugated to magnetic beads (Dynabeads). After extensive washing, the immuno-isolates were analyzed by gel electrophoresis and immunoblotting (Figure 10C). Immuno-isolates contained NR1 and NR2B, as expected. To characterize enrichment, we quantified the pixel intensities in the western blots and calculated the percentage of the total input protein that was recovered in the immuno-isolate fraction. We recovered 27.2 ± 7.8% of total NR1 and 18.9 ± 6.6% of total NR2B in the immuno-isolate (n = 7 experiments). Importantly, immuno-isolates were enriched for Nlgs (as determined using a pan-Nlg antibody; 15.2 ± 2.6% of input, n = 7). Analysis with antibodies specific to Nlgs 1, 2 and 3 revealed that all three Nlgs were present in these immuno-isolates (7.7 ± 1.7%, 3.9 ± 2.9% and 4.5 ± 3.1% of input, respectively; Figure 10C). Consistent with this idea, multiple studies suggest that Nlgs 2 and 3 localize to glutamatergic and GABAergic synapses [24,40,41]. However, some studies do suggest that Nlg2 is specifically associated with GABAergic synapses [42]. Nlg4 was not analyzed as it is not expressed at this time in development [29]. This suggests that multiple Nlgs may associate with NRTPs during trafficking to synapses.

We then analyzed the immuno-isolates for the presence of membrane-associated guanylate kinases (MAGUKs) that potentially mediate the Nlg-NMDAR interaction. Quantification of western blots demonstrated that SAP102, PSD-95 and S-SCAM were recovered in the immuno-isolate (6.6 ± 2.0%, 6.8 ± 1.6% and 5.3 ± 2.3% of total input, respectively; n ≥ 5; Figure 10C). This recovery was significant because the synaptic vesicle protein VGlut1 was not present in the NR1 immuno-isolated membranes (-1.9 ± 5.3%, *P *< 0.05, n = 4; Figure 10C). Although these experiments do not shed light on the stoichiometry of the proteins associated with NRTPs because labeling intensity is determined by antibody affinity, they are consistent with the conclusion from imaging and immunocytochemistry that NMDARs and Nlg1 are physically associated with each other and transported together in dendrites during synaptogenesis, presumably via interaction with a MAGUK protein.

In previous studies, we demonstrated that 28% of mobile NMDARs are associated with AMPARs [7]. Despite being unable to co-immunoprecipitate the AMPAR subunit GluR2 with an antibody to either NR2B or Nlg from detergent-solubilized brain (Figure 10B), we considered the possibility that GluR2 might also be present within our immuno-isolated NRTP membrane fraction. Indeed, western blotting and quantification demonstrated a significant enrichment of GluR2 in these fractions (18.2 ± 1.0% of input, n = 3; Figure 10C). This suggests that while there is no direct interaction between NMDARs and AMPARs or between Nlgs and AMPARs at this time in development (Figure 10B), AMPARs are present within immuno-isolated NRTPs. While the enrichment of AMPARs may appear stronger than that seen for any of the associated proteins, it is important to consider that these immuno-isolations are of intact membranes, which will include the AMPARs, whereas MAGUK proteins and Nlgs are peripherally associated (see below) and, hence, may be less efficiently co-isolated.

The biochemical association of Nlgs with NRTPs could occur through three possible mechanisms: Nlgs could be integrally associated within the NRTP membrane as a component of a multi-protein containing transport organelle; Nlgs could be indirectly associated with NRTPs through binding of NMDARs to a MAGUK, which in turn binds Nlgs [43-45]; or Nlgs could both be within NRTPs and associate with NMDARs. If Nlg1 is present in NRTPs, but not directly associated with NMDARs, then solubilization of the immuno-isolates with the detergent Triton X-100 should prevent recovery of Nlg1. However, solubilization of the immuno-isolates did not change enrichment of Nlg1 (Figure 10D). We recovered the same amount of NR1 in the detergent-solubilized conditions (100 ± 24% of the control condition, n = 4), and we recovered 79 ± 30% of Nlg1 in the presence of Triton X-100 (n = 4; Figure 10D). To confirm that the membranes were in fact being disrupted under these conditions, we examined GluR2 that was previously not co-immunoprecipitated under solubilizing conditions (Figure 10B). Consistent with this, only 15.3 ± 3.1% of GluR2 was retained in the NRTP immuno-isolate under solubilizing conditions compared to intact membranes (n = 2; Figure 10D). This result suggests that Nlg1 biochemically interacts with NMDARs and is not passively co-transported in the same organelles.

If Nlg1 is only peripherally associated with NRTPs, then a high pH wash of the immuno-isolated NRTPs should abolish this interaction since this treatment abrogates protein-protein interactions without compromising the integrity of the membrane-bound organelles [46]. In agreement with this interpretation, exposure of immuno-isolated NRTPs to high pH (carbonate buffer, pH11) eliminated enrichment of Nlg1 (6.1 ± 22% of the amount recovered at pH7.4, n = 4; Figure 10E). In contrast, both NR1 and GluR2 were significantly enriched under pH11 conditions (141 ± 47% and 66.4 ± 6.7% of the amount recovered at pH7.4, respectively, n = 4 and 2; Figure 10E). Together, these results suggest that Nlg1 is not contained within the NRTPs, but is associated with them, presumably via adapter proteins, such as SAP102, PSD-95 or S-SCAM.

### Co-transport of Nlg1 with NMDARs requires the Nlg1 PDZ binding motif

**Figure 11:**
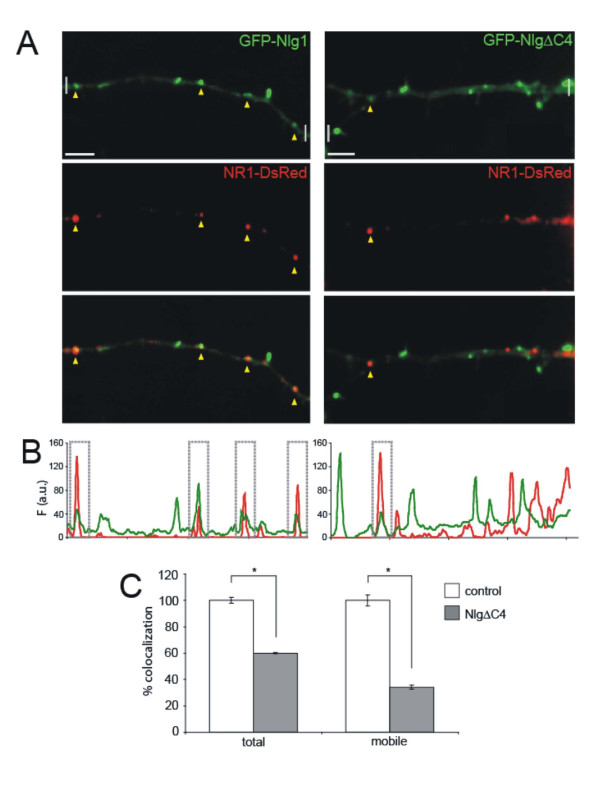
**Nlg PDZ binding motif is important for co-transport of Nlg1 and NMDA receptors**. **(A) **Time-lapse imaging of neurons co-transfected with either GFP-Nlg1 (green, left panel) and NR1-DsRed (red), or GFP-NlgΔC4 (green, right panel) and NR1-DsRed (red). Arrowheads indicate colocalized Nlg1 and NR1 clusters. Scale bar, 5 μm. **(B) **Fluorescence intensity profiles (in arbitrary units (a.u.)) along a line drawn through the dendrite, between the white faded lines shown in (A, top panels). Boxed regions around fluorescent intensity peaks correspond to colocalization of Nlg and NR1. **(C) **Deletion of the PDZ binding motif within the cytoplasmic tail of Nlg significantly reduced the colocalization of total and mobile Nlg clusters with NR1. Error bars are standard error; *P ≤ 0.001.

To test the hypothesis that co-transport of Nlg and NMDARs requires the PDZ domain of the cytoplasmic tail of Nlg1, 3–5 d.i.v cortical neurons were co-transfected with the Nlg deletion mutant, GFP-Nlg1ΔC4, lacking the PDZ binding domain, together with NR1-DsRed and imaged 12–14 hours later (Figure 11A, B). Consistent with our hypothesis, a 40% reduction in colocalization was observed between Nlg clusters and NR1 (from 58.5 ± 1.3% to 35.1 ± 0.3%, n = 8 and 15 neurons respectively, *P *= 0.0053; Figure 11C). Time-lapse fluorescent imaging also revealed a 66% reduction in the colocalization of mobile Nlg1 clusters with NR1-DsRed (from 61.6 ± 2.5% to 21.1 ± 1.0%, n = 6 neurons each, *P *= 0.001; Figure 11C). Although a small proportion of Nlg1 and NMDAR colocalization and co-transport might be mediated by additional parts of the cytoplasmic tail of Nlg1, this could not be tested due to the requirement of this domain for Nlg clustering (Figure 1E). Overall, these results indicate that the PDZ binding motif contained within the cytoplasmic tail of Nlg1 specifically mediates most of the colocalization and co-transport of Nlg1 with NMDARs, but is not crucial for the localization or mobility of Nlg itself [33] (Figure 1).

## Discussion

The cell adhesion molecule pair Nlg and NRX appears to be sufficient for the recruitment of two essential components of the PSD – NMDARs and PSD-95 [19,20,23]. It was thus proposed that a ternary complex between Nlg1, PSD-95 and NMDARs could directly mediate NMDAR recruitment to nascent synapses [32], since PSD-95 can bind directly to both Nlg1 [31] and to NMDARs [10,11]. However, this hypothesis is difficult to reconcile with data showing that the synaptic accumulation of PSD-95 occurs with a variable time course relative to NMDAR recruitment. In some cases, PSD-95 accumulation precedes NMDAR recruitment [7,8,17,47], while in other cases, NMDARs are recruited to nascent synapses in the absence of PSD-95 [7,48]. Importantly, NMDARs do not require PSD-95 for recruitment to new synapses [7,48], NMDARs do not require the PDZ binding motif of Nlg to localize to synapses [24], and Nlg does not require its carboxy-terminal PDZ domain to be targeted to synapses [33]. Here, we have shown that Nlg1 can recruit multiple postsynaptic proteins to nascent synapses via independent mechanisms and time courses.

In young cortical neurons prior to and during synaptogenesis, Nlg1 is present in at least four distinct surface pools: diffuse; immobile clustered; motile clustered; and motile clustered associated with NMDARs. It is unclear how much exchange there is between the pools of surface Nlg1, but our imaging is consistent with the possibility that axonal contact leads to clustering of diffuse Nlg at nascent synapses (Figure 2). Analogously, the diffuse pool can be made to aggregate into patches by applying a soluble form of its ligand β-NRX. The immobile clusters of Nlg1 may represent a pool of Nlg1 already associated with PSD-95, which has been shown to passively recruit presynaptic terminals [13]. The motile pools of Nlg comprise approximately 11% of Nlg clusters, being transported within dendrites at an average speed of approximately 15 μm/minute. Mobile Nlg clusters were often seen in dendritic filopodia, placing Nlg in a perfect position to mediate and stabilize new axodendritic contacts [49]. The kinetics of Nlg cluster movement that we have measured differ from what has been reported previously [13]. Our higher mobility may be explained by the age of the culture (4 versus 7 days), by the fast rate of imaging in this study (up to 2 frames per second compared to 2 frames per minute) or by the source of the neuronal cultures (cortex versus hippocampus). Regardless of specific differences, both reports are consistent in showing that Nlg clusters are mobile and, thus, could be recruited to nascent synapses.

Accumulation of GFP-Nlg1 occurred rapidly following axodendritic contacts in young cortical neurons. In the majority of examples, Nlg1 accumulated at nascent contacts of axonal growth cone filopodia with dendritic shafts in just over 1 minute of stabilized contact; this is the first direct demonstration of Nlg1 accumulation at new contacts between axons and dendrites, and provides evidence for the prevailing assumption in our field that accumulation of a CAM is one of the first steps following axodendritic contact leading to the formation of a nascent synapse [1,6]. Given the timing of just 1 minute, it is tempting to speculate that this event might be associated with the calcium transients recently described during axodendritic contact events that are stabilized in intact tissue [50]. The remaining third of our examples showed a slower accumulation of Nlg1 within 8.1 minutes, on average, which is precisely the average time course of NMDAR recruitment to these kinds of contacts [7]. Finally, Nlg1 clusters can also be recruited to existing synapses after their formation, perhaps providing additional stabilization through increased adhesion and/or recruitment of more NMDARs. Taken together, these data suggest that Nlg1 may accumulate at nascent contacts within seconds to enhance adhesion. Within minutes of stabilization of the contact, additional Nlg1 is recruited that may bring NMDARs directly to the contact site and begin the process of synaptogenesis.

Because visualizing the recruitment of tagged proteins to axodendritic contacts is prohibitively low-yield, we developed an assay to reliably and rapidly cluster Nlg in the dendritic membrane of young, cultured cortical neurons. The advantage of this assay is that it can reveal the effects of oligomerization of endogenous or recombinant Nlg on recruitment of endogenous postsynaptic proteins with high temporal resolution. Nlg oligomerization in young cortical neurons before the peak of synaptogenesis dramatically increased PSD-95 clustering. This result is consistent with previous reports demonstrating that PSD-95 is recruited to sites of contact with β-NRX presented on either transfected non-neuronal cells [20] or coated onto beads [19,23]. However, in contrast to those previous studies, we used time-lapse imaging to characterize a more precise timing and mechanism of PSD-95 recruitment to new Nlg1 clusters. Because β-NRX-Nlg binding is potentially one of the first events that leads to the rapid accumulation of postsynaptic proteins to nascent synapses within minutes of contact between axons and dendrites [1,45], we specifically investigated the time course of recruitment of PSD-95 to oligomerized Nlg using our patching assay. PSD-95 accumulated at Nlg clusters between 30 and 60 minutes after patching, a time course consistent with that seen for accumulation of PSD-95 at synapses between neurons in culture [7,12,17,23,47,51]. Recruitment of PSD-95 to Nlg patches was completely dependent on the PDZ domain of Nlg and on palmitoylation, similar to its recruitment to synapses [36]. Given the strong influence of PSD-95 on AMPAR recruitment to new synapses [36], a delay of between 30 and 60 minutes for PSD-95 arrival is also consistent with the arrival of AMPA receptors, which occurs 1–2 hours after synaptic contact [7,17,23].

In contrast to the dramatic increase in density of PSD-95 clusters, the density of NMDAR clusters was unchanged following Nlg patching. This result was surprising since previous reports showed that NMDARs are recruited to sites of contact with non-neuronal cells expressing β-NRX [20] and beads coated with β-NRX within 2–4 hours [19,23]. Given those previous results, we initially expected that Nlg patching might increase clustering of NMDARs. However, because NMDARs are present in NRTPs, their recruitment would not be seen as an increase in number upon patching of Nlg1. Of course, it is possible that new NMDAR clusters appear with a longer time course after Nlg patching, but Nlg patching clearly does not lead to the formation of new NMDAR clusters within the 10 minute to 1 hour time-frame in which NRTPs are recruited to nascent synapses [7].

Although Nlg patching did not increase the density of NMDAR clusters, Nlg oligomerization did increase the intensity of NMDAR staining at existing synapses in a palmitoylation-dependent manner, suggesting that Nlg increases recruitment of NMDARs to established synapses via an interaction with a palmitoylated protein, presumably PSD-95. This result is consistent with the observation that the PDZ domain of Nlg is required for Nlg-mediated development of NMDAR currents at synapses [20], and Nlg1 is required for a late-stage activity-dependent maturation of NMDAR currents [28].

Perhaps the most surprising result in this study was that NMDARs are highly colocalized with Nlg clusters before synapses are formed and mobile NRTPs have a high propensity to travel with Nlg clusters. Approximately 70% of mobile Nlg1 clusters are co-transported with NRTPs. NRTPs can interact stably or transiently with Nlgs 1, 2 and 3 during transport and patching Nlgs dramatically reduces their mobility. While we do not yet know the precise molecular links between surface Nlgs and NRTPs, our results are consistent with the hypothesis that this trafficking interaction is mediated by scaffolding proteins such as SAP102, S-SCAM or PSD-95. First, these scaffolding proteins are present on the immuno-isolated NMDAR-containing organelles and bind both NMDARs [10,11,52-54] and Nlg1 [31,43,44]. Second, mobile NRTPs are highly colocalized with SAP102 [9] and Nlg1 (Figure 7) and NMDARs are strongly associated with SAP102 in young brain [54]. Perhaps most compelling, deletion of the carboxy-terminal PDZ domain (ΔC4) dramatically decreased the colocalization and co-transport of Nlg1 and NMDARs, without affecting the clustering or mobility of Nlg1 alone. The region between the last 55 and 4 amino acids containing the WW domain that binds S-SCAM [43] is sufficient for clustering and transport of Nlg1 and may mediate the small amount of colocalization and co-transport of Nlg1 and NMDARs that remains in neurons transfected with PDZ domain-lacking Nlg1(ΔC4). This compensation by the WW domain would explain the apparent normal recruitment of NMDARs in the presence of the ΔC4 mutant Nlg1 during long-term overexpression experiments [24].

The mobility of Nlg clusters and co-transport of Nlg1 with NRTPs was particularly surprising since Nlg is primarily found in the plasma membrane of young cortical neurons (Figure 1A). Using both acid-stripping and biotinylation assays, we have previously shown that surface Nlg does not undergo endocytosis or cycling with the plasma membrane within 30 minutes of antibody-binding in young cortical neurons [9]. Surface-labeling experiments here further confirm the surface localization of Nlg1 and show that patching using either GFP antibodies or β-NRX does not induce internalization of Nlg1 (Additional file 1D). In contrast, NMDARs cycle with the plasma membrane during their transport on heterogeneous tubulovesicular structures that move intracellularly along microtubules [9]. Together, these results imply that surface clusters of Nlg are co-transported with cycling and intracellular NRTPs. The mechanisms underlying this co-transport are not known and will be an important topic for future work.

**Figure 12:**
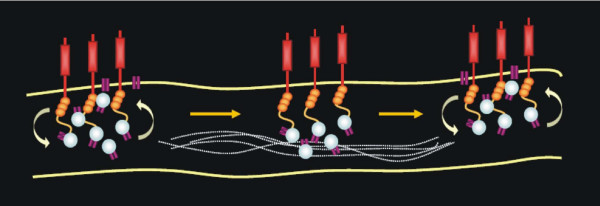
**Model of the association between Nlg and NMDA receptor transport packets (NRTPs) during transport. NRTPs cycle with the plasma membrane (pale arrows) during pauses of microtubule-dependent transport (yellow arrows)**. These can be associated with clusters of Nlg (red) at the membrane both during transport and pausing. The interaction between surface Nlg and NRTPs is likely via membrane-associated guanylate kinases (orange beads on a string).

The observation that a surface CAM can be trafficked with an intracellular transport organelle is uncommon, although not unprecedented. Indeed, surface NCAM forms a complex with NMDARs in young neurons through a spectrin linkage; this interaction of NRTPs with NCAM is important for recruitment of NMDARs to nascent synapses [55]. Presynaptically, surface NCAM also associates through spectrin with intracellular trans-Golgi network organelles [46] and surface TrkB is co-transported with synaptic vesicle precursors [56]. Here, we have shown that surface Nlg1 is co-transported with NRTPs, presumably through binding to several possible MAGUKs, including SAP102 or S-SCAM. Taken together, these results suggest a novel model for CAM function: surface CAMs can be linked via cytosolic scaffolding proteins to intracellular transport organelles (Figure 12). Oligomerization of the surface CAM, or binding of that CAM to its partners on a presynaptic axon, would then lead to the accumulation of the synaptic protein directly linked to the CAM. Consistent with this idea, Nlg patching decreases NRTP motility, suggesting that tethering of Nlg1 by a presynaptic terminal would cause NR1 to pause and/or stop at a new site of contact through its physical association with Nlg1. This model of Nlg1-dependent localization of NMDARs to new sites of synapse formation would predict decreased NMDAR function in Nlg1-deficient rodents. Indeed, NMDAR-mediated synaptic transmission is decreased in Nlg1 knockout mice [29] and NR1 expression is decreased by 23% in Nlg triple knockout mice (Nlgs 1, 2 and 3) [29]. Furthermore, Nlg1 knock-down in the amygdala of rats results in decreased associative fear memory, an NMDAR-dependent behavior [57].

## Conclusion

Nlg1 accumulates at nascent axodendritic contacts with a rapid time course, placing it at the right place and time to strengthen adhesion and recruit proteins to the PSD. Nlg oligomerization recruits two different postsynaptic proteins – NMDARs and PSD-95 – with distinct mechanisms and time courses. This demonstrates that Nlg1 is sufficient for *de novo *and rapid PSD formation (but see [28]). NMDARs are recruited to sites of Nlg oligomerization with a very fast time course [7] through a physical interaction with Nlg, likely mediated by a PDZ-containing protein such as SAP102. Accumulation of PSD-95 at these sites occurs with a slower time course and is palmitoylation-dependent. Later, additional packets of NMDARs can be added to established synaptic sites via a Nlg-dependent recruitment of NMDARs associated with PSD-95 and requiring palmitoylation. Thus, rapid accumulation of Nlg1 at nascent synapses appears to be pivotal in orchestrating multiple independent steps in the formation of glutamatergic synapses.

## Materials and methods

All studies were conducted with approved protocols from both the University of California Davis Animal Care and Use Committee and the University of Oregon Animal Care and Use Committee, in compliance with NIH guidelines for the care and use of experimental animals.

### DNA constructs

CFP-Nlg1 was made by excising the *Sal*I GFP fragment from GFP-Nlg1 and inserting CFP amplified with the same primers as used previously for GFP-Nlg1 [32]. GFP refers to the enhanced green fluorescent protein (EGFP). Deletion of the carboxy-terminal 4 (ΔC4) and 55 (ΔC55) amino acids of Nlg1 was performed by PCR and confirmed by sequencing. NR1-DsRed was described previously [7]. pCDNA-β-NRX-Fc was a gift from P Scheiffele (Biozentrum, Basel, Switzerland). PSD-95-GFP was a gift from Alaa El-Husseini.

### Neuronal culture and transfections

Neurons from rat cortex were cultured and plated on astrocyte monolayers at a density of 22 K/18 mm coverslip [7]. Neurons were transfected with Lipofectamine 2000 (Invitrogen, Carlsbad, California, USA) as described previously [9]. Data were collected from at least two separate neuronal cultures for all experiments.

### Patching experiments

For patching of endogenous Nlg, a fusion protein of the extracellular domain of β-NRX and human IgG Fc was produced by transfecting COS7 cells with pCDNA-β-NRX-Fc. The medium was passed through a Protein-G-sepharose column (Amersham, Piscataway, NJ, USA) and eluted with glycine buffer pH2.7 and concentrated to approximately 1 mg/ml. β-NRX-Fc (0.01 mg/ml) was pre-incubated with anti-human IgG antibodies (1:250; Chemicon, Temecula, California, USA) in neuronal medium for 15 minutes at room temperature. Neurons were incubated with this medium for various periods, rinsed with artificial cerebrospinal fluid [7] and then imaged live or fixed for immunocytochemistry. For patching of GFP/CFP-Nlg1, polyclonal anti-GFP antibodies (1:750; Molecular Probes, Eugene, Oregon, USA) were pre-incubated with anti-rabbit secondary antibodies (1:3,000; Molecular Probes) in neuronal medium for 15 minutes at room temperature. Neurons were then incubated with this medium for various periods, rinsed with artificial cerebrospinal fluid and imaged or processed.

### Immunocytochemistry

Immunolabeling with anti-NR1 antibody (54.1, 1:500; BD Biosciences PharMingen, San Diego, California, USA) was carried out as described [7] using methanol at -20°C for 10 minutes as a fixative. All other immunocytochemistry used 4% paraformaldehyde, 4% sucrose in phosphate-buffered saline at 4°C for 10 minutes as a fixative. Coverslips were exposed to 0.25% Triton X-100 for 5 minutes, blocked with 10% bovine serum albumin in phosphate-buffered saline and incubated with primary and secondary antibodies in 3% bovine serum albumin. Primary antibodies used were as follows: Nlg1 (4C12, an antibody specific for Nlg1, 1:400; N Brose), Nlg (4F9, 1:500; Synaptic Systems, Gottingen, Germany), PSD-95 (K28/43, 1:800; J Trimmer), VGlut1 (AB5905, 1:2,000; Chemicon, Temecula, California, USA), NR2A (07-632, 1:500; Upstate Biotechnology, Lake Placid, New York, USA), NR2B (AB1557, 1:200; Chemicon), GFP (A11122, 1:2,000; Molecular Probes), and GFP (A11120, 1:500; Molecular Probes). Secondary antibodies used were Alexa-fluor conjugated anti-rabbit, anti-mouse, anti-guinea pig and anti-human (Molecular Probes).

### Imaging

Live-imaging was conducted in an imaging chamber for 18 mm coverslips (QE-1; Warner, Hamden, Connecticut, USA) on an Eclipse TE300 Nikon inverted microscope using a 60× oil immersion objective (1.4 NA). Fluorophores were excited at their absorption maxima using a TILL Photonics (Martinsried, Germany) monochromator combined with double and triple band pass filters specific for CFP-DsRed and FITC-Texas Red-Cy5 (Chroma, Battleboro, Vermont, USA). Bleed-through from chromophores into other channels was tested by using only one chromophore and checking all other wavelength and filter combinations for detectable signal as previously described [9]. Images were acquired sequentially with a CoolSNAP HQ CCD camera (Roper Scientific, Tucson, Arizona, USA) and Simple PCI software (C-Imaging, Compix Inc., Cranberry Township, Pennsylvania, USA). For contact experiments, live imaging was conducted on either an Eclipse TE 300 Nikon inverted microscope with a 60× oil immersion objective (1.45 NA) with filter wheel excitation and emission (Sutter Instrument, Novato, California, USA) or a Zeiss (Thornwood, New York, USA) Pascal confocal system with a 60× oil immersion objective (1.4 NA). Imaging was conducted with continual perfusion of artificial cerebrospinal fluid from a gravity fed perfusion system. Images were typically collected at 25 s intervals, as this provided the best compromise between high temporal resolution and minimizing neuronal toxicity and photobleaching. For imaging of immunocytochemistry, coverslips were viewed with either an Olympus Fluoview 2.1 laser scanning confocal system with a 60× PlanApo oil immersion objective (1.4 NA) on an IX70 inverted microscope or a Nikon C1 laser scanning confocal system with a 60× PlanApo water immersion objective (1.2 NA) on a Nikon TE 2000-U. Images for each chromophore were acquired sequentially and averaged over at least two scans.

### Immunoprecipitation of Nlg and immuno-isolation of NRTPs

For immunoprecipitation, the cortices of P4 rat pups were homogenized in 50 mM Tris-HCl pH7.4 in a Dounce-Elvehjem homogenizer. Membranes were pelleted by centrifuging at 100,000 × g for 30 minutes at 4°C. The pellet was resuspended in 50 mM Tris-HCl, 1 mM EDTA pH9. Membranes were solubilized with deoxycholate 1% for 45 minutes at 37°C. Insoluble material was removed by centrifuging at 100,000 × g for 60 minutes at 4°C. Triton X-100 was added to 0.1% and the extracts were then dialyzed overnight against 50 mM Tris-HCl, pH 7.5, 0.1% Triton X-100 at 4°C. Protein (1 mg) was incubated with 10–15 μl of Nlg or NR2B antibody overnight, Protein G sepharose beads (Sigma, St. Louis, Missouri, USA)) were added for 1 hour. Beads were washed with the dialysis buffer and then boiled in Laemmli sample buffer and submitted to western blotting and labeling with antibodies (see details below).

For immuno-isolation, the cortices from two rat pups (P2–3) were homogenized in homogenization buffer (4 mM HEPES (pH 7.4), 0.32 M sucrose with protease inhibitors (Roche, Indianapolis, Indiana, USA))), using eight strokes of a Dounce-Elvehejm homogenizer. The homogenate was centrifuged for 10 minutes at 1,000 × g at 4°C. The supernatant was re-centrifuged for 15 minutes at 20,000 × g at 4°C. Dynabeads (1 × 10^8^) that had been preabsorbed with anti-NR1 antibody per company instructions (Invitrogen, Carlsbad, California, USA) were incubated with 250 μg of the resulting rat brain supernatant in the presence of 5% bovine serum albumin overnight at 4°C. The beads were washed extensively with the homogenization buffer and then resuspended in Laemmli sample buffer. For solubilization, one of the washes was carried out with 0.25% TritonX-100 for 30 minutes. For the high pH wash, the beads were incubated with carbonate buffer pH11 for 30 minutes. The samples were submitted to western blotting and labeling with the following antibodies: anti-NR1 (54.1, 1:500; BD Biosciences Pharmingen), anti-NR2B (Ab1557, 1:500; Upstate Biotechnology), anti-PSD-95 (K28/43, 1:2,000; JS Trimmer), anti-SAP102 (1:1,000; R Wenthold), anti-S-SCAM (1:1,000; Y Hata), anti-VGlut1 (AB5905, 1:1,000; Chemicon), anti-Nlg (4F9, 1:1,000; Synaptic Systems), anti-Nlg1 (4C12, 1:1,000; Synaptic Systems), anti-Nlg2 (1:1,000; A El-Husseini), anti-Nlg3 (1:1,000; A El-Husseini), anti-synapsin1 (AB1543, 1:1,000; Chemicon) and anti-GluR2 (MAb397, 1:800; Chemicon).

### Data analysis

Quantification of immunocytochemistry and colocalization during live imaging was performed as detailed previously [7] using the raw images in Image-Pro Plus software, but with the following modifications (Media Cybernetics, Bethesda, Maryland, USA). Intensities were measured within approximately 1 μm diameter circles around the center of manually defined clusters in each channel. The threshold used to subtract background fluorescence was the mean dendritic background intensity plus one standard deviation. One standard deviation was generally on the order of 1.3-fold the mean dendritic background. Quantification of transport velocities was determined by measuring the time from the start to the end of a unidirectional motion to give mean velocity. Clusters were considered mobile when they performed a unidirectional movement of >2 μm across at least three successive images. Quantification of western blots was carried out using ImageJ (NIH). Intensity of control samples was subtracted from NR1 immuno-isolated samples and this was expressed as a percentage of total input. Statistics were analyzed in Excel using a two-tailed Student's *t*-test. Images were processed post-quantification using Adobe Photoshop for presentation purposes.

## Abbreviations

AMPA: α-amino-3-hydroxy-5-methyl-4-isoxazolepropionic acid; AMPAR: AMPA receptor; CAM: cell adhesion molecule; CFP: cyan fluorescent protein; CNS: central nervous system; d.i.v.: days *in vitro*; GFP: green fluorescent protein; MAGUK: membrane-associated guanylate kinase; Nlg: neuroligin; NMDA: N-methyl-D-aspartic acid; NMDAR: NMDA receptor; NR1: NMDAR subunit 1; NR2A/B: NMDAR subunit 2A/B; NRTP: NMDAR transport packet; NRX: neurexin; PSD: postsynaptic density; SAP: synapse-associated protein; S-SCAM: synaptic scaffolding molecule; VGlut: vesicular glutamate transporter.

## Competing interests

The authors declare that they have no competing interests.

## Authors' contributions

AKM and PW conceived the design of this study and wrote most of the manuscript. PW performed many of the live imaging experiments, mostly in the McAllister laboratory with more recent experiments imaged in the Washbourne laboratory. SLB and EC performed some of the live-imaging and immunocytochemical experiments in the McAllister laboratory. JRLC performed many of the control experiments in the Washbourne laboratory. FE-S performed the biochemical characterization of NRTPs in the McAllister laboratory.

## Supplementary Material

Additional File 1 **Expression of GFP-Nlg1 does not change endogenous Nlg distribution**. **(A) **Primary neurons in culture expressing recombinant Nlg1 and neighboring non-transfected neurons (red only). Twelve to 14 hours of expression of recombinant Nlg1 did not change the density of Nlg puncta in the dendrites of transfected compared to non-transfected neurons (0.84 ± 0.05 puncta/20 μm for transfected neurons versus 0.82 ± 0.2/20 μm in non-transfected cells, *P *> 0.9, n = 7 neurons). However, GFP-Nlg1 expression did increase the overall intensity of diffuse and punctate dendritic Nlg 2.4-fold (± 0.23) and 2.6-fold (± 0.28; *P *< 0.01, n = 7 neurons), respectively, in transfected versus neighboring non-transfected neurons. The ratio between cluster intensity and mean dendritic intensity was unchanged between transfected and non-transfected neurons (0.95 ± 0.18 and 0.86 ± 0.15, respectively; *P *> 0.6), suggesting that the diffuse versus clustered distribution was unaffected by overexpression. Furthermore, the numbers of synapses formed by transfected and non-transfected cells was the same (0.84 ± 0.15 and 0.88 ± 0.2 synapses/20 μm, *P *> 0.7, n = 16), presumably due to the short expression time (12–14 h). A similar distribution of Nlg1 was reported using an alternative surface-labeling protocol employing monomeric avidin [30]. **(B) **Neurons transfected with GFP-Nlg1 and incubated with anti-GFP antibody (patched) showed increased punctate localization of the GFP-Nlg1. **(C) **The increase in the number of non-synaptic GFP-Nlg1 clusters reached its maximum already after 30 minutes. **(D) **Patching of GFP-Nlg1 (green) with β-NRX-Fc (blue) did not cause significant internalization, because 89.5 ± 4.7% of GFP clusters were still labeled with anti-GFP antibody (red) applied to non-permeabilized, live neurons (n = 6 neurons). Scale bar, 5 μm. **(E) **Surface labeling of GFP (red) did not permeabilize live neurons as shown by the lack of staining for the intracellular dendritic marker MAP2 (blue). In contrast, application of these two antibodies to fixed and permeabilized cells revealed labeling for both GFP and MAP2. Scale bar, 20 μm. Click here for file

Additional File 2 **Additional examples of GFP-Nlg1 accumulation at nascent axodendritic contacts**. **(A) **Time-lapse imaging of the formation of a contact between the dendrite of a 5 d.i.v. neuron transfected with GFP-Nlg1 and an axon of a non-transfected neuron, visualized with transmitted light. Fluorescent images of the dendritic region highlighted by the dotted white boxed region are shown to the right. Time 0:00 is defined as the point of initial contact; the contact site is indicated by an asterisk. Nlg1 accumulated at this contact site, as shown by white arrowheads, within minutes. Scale bar, 5 μm. **(B) **Time-lapse imaging of a GFP-Nlg1 transfected neuron during the formation of another axodendritic contact. The axon (not visible) is identified by a dotted outline on each image for clarity. Time 0:00 is the time of initial contact. Extension of a dendritic filopodia along the contacting axon occurred shortly after contact, with Nlg1 clustered at the filopodial tip (black arrowhead). The filopodium remained extended along the axonal growth cone for the rest of the imaging period. Scale bar, 5 μm. Click here for file

Additional File 3 **Accumulation of Nlg1 at a site of axodendritic contact**. Time-lapse movie of the formation of a contact between the dendrite of a 5 d.i.v. neuron transfected with GFP-Nlg1 and an axon transfected with mCherry for visualization (from Figure 2A, C). Nlg1 accumulated at the site of axodendritic contact within 50 s, followed by extension of a filopodia from the main dendritic branch. Total imaging time is 1 hour and 3 minutes. Click here for file

Additional File 4 **Co-transport of Nlg1 with NRTPs**. Time-lapse movie of a cortical neuron in culture at 4 d.i.v transfected with CFP-Nlg1 (green) and NR1-DsRed (red) demonstrates colocalization of these two membrane proteins during trafficking in dendrites (yellow motile clusters). Total imaging time is 28 minutes and 35 s. Click here for file
