# Supplementary figures and images for: Neuroligin1: a cell adhesion molecule that recruits PSD-95 and NMDA receptors by distinct mechanisms during synaptogenesis
Source: Neural Dev. 2009 May 18;4:17. doi: 10.1186/1749-8104-4-17 (PMC2694798; doi:10.1186/1749-8104-4-17)

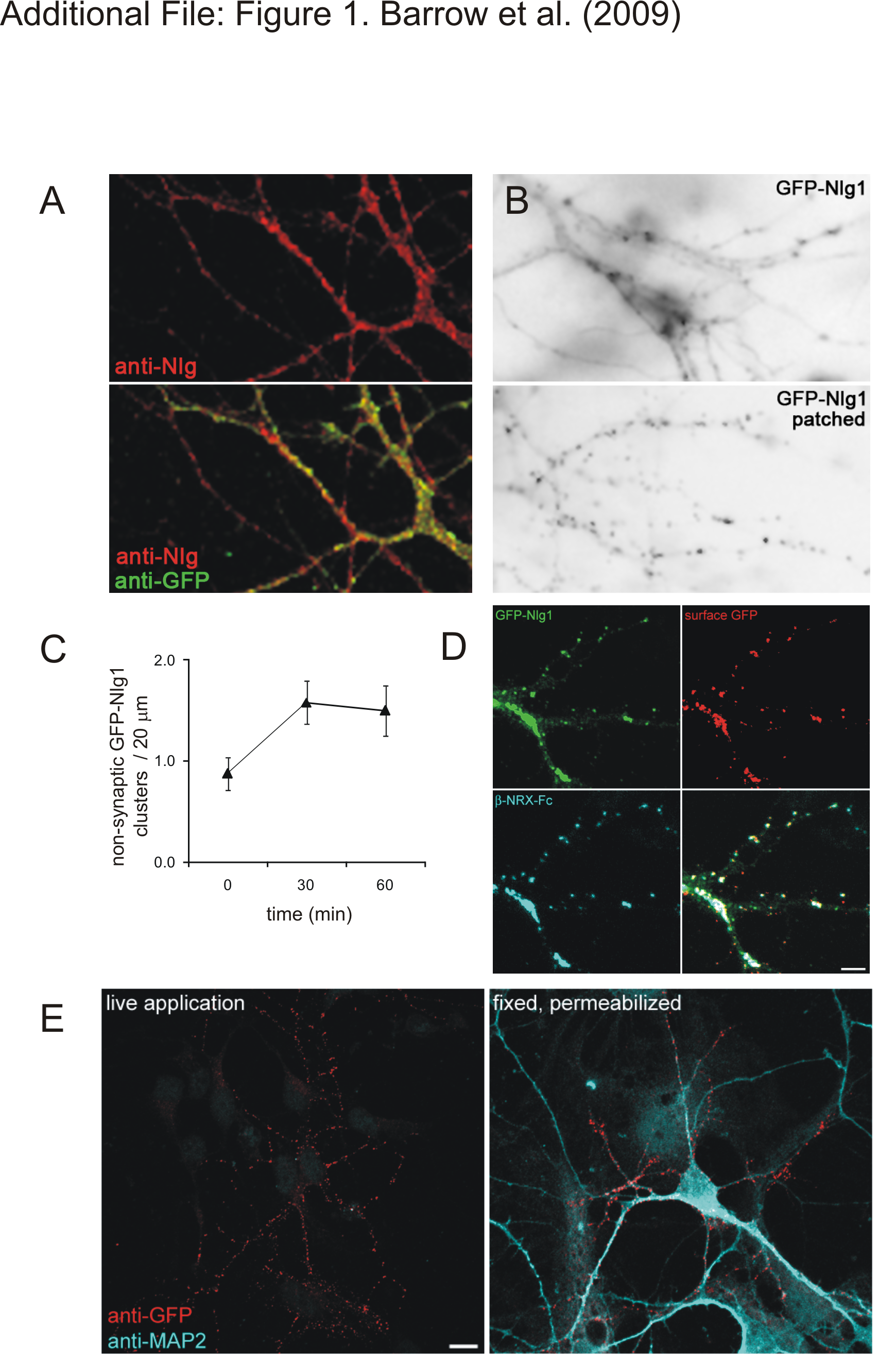

Supplement: Additional File 1 — Expression of GFP-Nlg1 does not change endogenous Nlg distribution. (A) Primary neurons in culture expressing recombinant Nlg1 and neighboring non-transfected neurons (red only). Twelve to 14 hours of expression of recombinant Nlg1 did not change the density of Nlg puncta in the dendrites of transfected compared to non-transfected neurons (0.84 ± 0.05 puncta/20 μm for transfected neurons versus 0.82 ± 0.2/20 μm in non-transfected cells, P > 0.9, n = 7 neurons). However, GFP-Nlg1 expression did increase the overall intensity of diffuse and punctate dendritic Nlg 2.4-fold (± 0.23) and 2.6-fold (± 0.28; P < 0.01, n = 7 neurons), respectively, in transfected versus neighboring non-transfected neurons. The ratio between cluster intensity and mean dendritic intensity was unchanged between transfected and non-transfected neurons (0.95 ± 0.18 and 0.86 ± 0.15, respectively; P > 0.6), suggesting that the diffuse versus clustered distribution was unaffected by overexpression. Furthermore, the numbers of synapses formed by transfected and non-transfected cells was the same (0.84 ± 0.15 and 0.88 ± 0.2 synapses/20 μm, P > 0.7, n = 16), presumably due to the short expression time (12–14 h). A similar distribution of Nlg1 was reported using an alternative surface-labeling protocol employing monomeric avidin [30]. (B) Neurons transfected with GFP-Nlg1 and incubated with anti-GFP antibody (patched) showed increased punctate localization of the GFP-Nlg1. (C) The increase in the number of non-synaptic GFP-Nlg1 clusters reached its maximum already after 30 minutes. (D) Patching of GFP-Nlg1 (green) with β-NRX-Fc (blue) did not cause significant internalization, because 89.5 ± 4.7% of GFP clusters were still labeled with anti-GFP antibody (red) applied to non-permeabilized, live neurons (n = 6 neurons). Scale bar, 5 μm. (E) Surface labeling of GFP (red) did not permeabilize live neurons as shown by the lack of staining for the intracellular dendritic marker MAP2 (blue). In contrast, appli [file 1749-8104-4-17-S1.png]

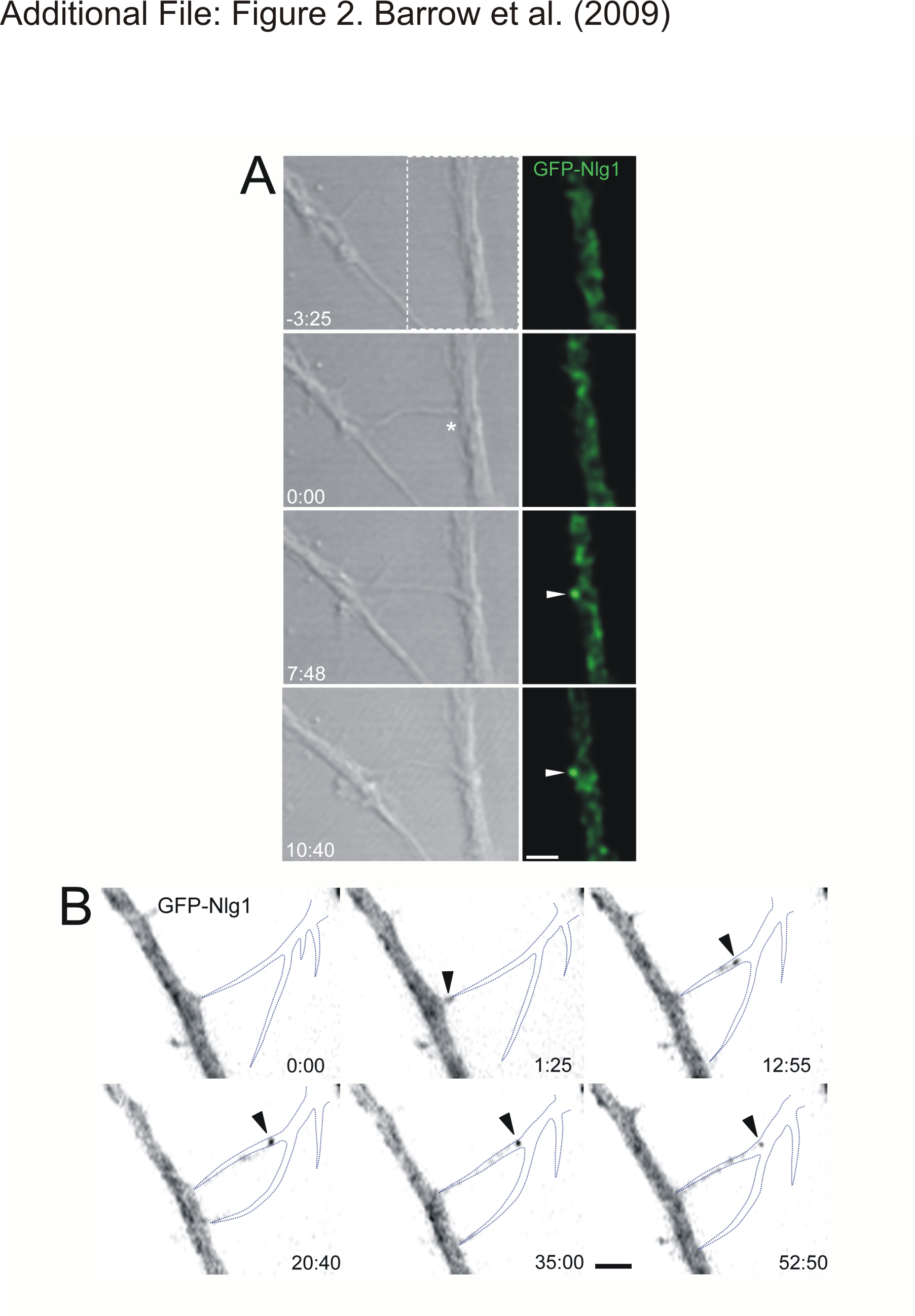

Supplement: Additional File 2 — Additional examples of GFP-Nlg1 accumulation at nascent axodendritic contacts. (A) Time-lapse imaging of the formation of a contact between the dendrite of a 5 d.i.v. neuron transfected with GFP-Nlg1 and an axon of a non-transfected neuron, visualized with transmitted light. Fluorescent images of the dendritic region highlighted by the dotted white boxed region are shown to the right. Time 0:00 is defined as the point of initial contact; the contact site is indicated by an asterisk. Nlg1 accumulated at this contact site, as shown by white arrowheads, within minutes. Scale bar, 5 μm. (B) Time-lapse imaging of a GFP-Nlg1 transfected neuron during the formation of another axodendritic contact. The axon (not visible) is identified by a dotted outline on each image for clarity. Time 0:00 is the time of initial contact. Extension of a dendritic filopodia along the contacting axon occurred shortly after contact, with Nlg1 clustered at the filopodial tip (black arrowhead). The filopodium remained extended along the axonal growth cone for the rest of the imaging period. Scale bar, 5 μm. [file 1749-8104-4-17-S2.png]
